# Derivative-Free Observability Analysis for Sensor Placement Optimization of Bioinspired Flexible Flapping Wing System

**DOI:** 10.3390/biomimetics7040178

**Published:** 2022-10-26

**Authors:** Bingyu Jin, Hao Xu, Jicheng Peng, Kelin Lu, Yuping Lu

**Affiliations:** 1 College of Automation Engineering, Nanjing University of Aeronautics and Astronautics, Nanjing 210016, China; 2 School of Automation, Southeast University, Nanjing 210096, China

**Keywords:** derivative-free observability analysis, stochastic system with memory, optimal sensor placement

## Abstract

Observability analysis of a bioinspired flexible flapping wing system provides a measure of how well the states of flexible flapping wing micro-aerial vehicles can be estimated from real-time measurements during high-speed flight. However, the traditional observability analysis approaches have trouble in terms of lack of quantitative analysis index, high computational complexity, low accuracy, and unavailability in stochastic systems with memory, including bioinspired flexible flapping wing systems. Therefore, a novel derivative-free observability analysis method is proposed here based on the generalized polynomial chaos expansion. By formulating a surrogate model to represent the relationship between the cumulative measurement and the random initial state, the observability coefficient matrix is calculated and the observability rank condition is stated. Consequently, several observability indices are proposed to quantity the observability of the system. Altogether, the proposed method avoids the disadvantages of the traditional approaches, especially in assessing the observability degree of each state and the effect of stochastic noise on observability. The validation of the proposed method is first provided by demonstrating the equivalence between the traditional and proposed methods and subsequently by comparing the observability of the Lorenz system calculated via three different approaches. Finally, the proposed method is applied on a bioinspired flexible wing system to optimize the placement of sensors, which is consistent with the natural configuration of campaniform sensilla on the wing of the hawkmoth.

## 1. Introduction

Due to the high maneuverability and portable structure of birds and insects, bioinspired flexible flapping wing micro-aerial vehicles (FWMAVs) have attracted considerable attention in the last decade [1,2,3]. A prerequisite to designing such agile FWMAVs is the simultaneous and accurate estimation of vehicle states, such as position and rotation rates, through the noisy information measured by the sensors on the wing. To solve this state estimation problem, the observability analysis of a bioinspired flexible flapping wing system is necessary in order to evaluate whether the system states can be inferred from the measurements. Thereby, the guidelines for measurement selection, sensor placement, and model optimization can be provided.

For a bioinspired flexible flapping wing system, deformation inevitably happens because of aerodynamic loads and inertial forces, and negatively influences the estimation of rotation rates based on the measurements from sensors on the wing. In nature, insects have sensing mechanisms that enable them to understand their flying states. For example, the strain information of a flying insect is measured and saved by campaniform sensilla on the wing, followed by conversion to action potentials (voltage spikes) in the sensory neurons [4,5]. After the delivery of these neural-encoded spikes to the central nervous system, the rotation rates are detectable. Such sensing and signal processing mechanics ensure insects’ fast and accurate sensory feedback and subsequent high agility [6]. Inspired by these biological mechanisms, the neural encoding processing of strain data can be constructed as neural encoding measurement model [7]. Unlike the raw strain measurement model used previously [8], the neural encoding measurement model has strong nonlinearity, the same as the flexible wing flapping dynamics model. In addition, the measurement at each time contains the current and previous state information. Altogether, the strong nonlinearity and memory function of the flexible flapping wing system make its observability analysis a significant challenge.

For a linear time-invariant system, the traditional observability rank condition is the simplest and most effective criterion to analyze whether the system is observable or not [9]. However, it fails in nonlinear systems, and even in linear time-variant systems. Hence, several observability analysis approaches for nonlinear systems have been developed. Rouhani et al. [10] applied the Lie-derivative-based observability analysis method to power systems with the realistic synchronous generator model, and Seo et al. [11] used the same method to analyze the observability of a missile interception system with line-of-sight angle measurement. This method is feasible only for simple and small-scale nonlinear systems, as it requires considerable derivation calculations. The empirical observability Gramian method, which is developed from the observability Gramian method through interval approximation, is more prevalent in practical applications because it does not depend explicitly on the system models and reduces the computational complexity [12]. For instance, Hinson et al. [8] utilized this method to determine the optimal sensor placement on the wing of the hawkmoth. Hodzic et al. [13] and Hinson et al. [14] respectively applied the empirical observability Gramian method to the sensor placement problem for generic wide-body aircraft and the path planning problem for under-sensed vehicles. However, the existing approaches are either derivative-based, which leads to high computational complexity, or approximation-based, which may reduce the analytical accuracy. Furthermore, these approaches are unable to measure the influence of stochastic noise on observability.

Recently, a derivative-free approach based on the generalized Polynomial Chaos (gPC) expansion has been proposed by Zheng et al. [15] to address the aforementioned problems. They further applied the derivative-free approach to a power system using the stochastic dynamic model [16]. While this method has been proven feasible and computationally efficient for nonlinear systems without control input and memory, it is impracticable for systems with memory, such as a flexible flapping wing system.

In this paper, we propose a modified gPC-based observability analysis approach for both linear and nonlinear systems incorporating memory and applying it to the analysis of a flexible flapping wing system. The observability indices obtained by the proposed approach are used to determine the optimal placement of sensors configured on the wing. The main contributions are as follows.

(1) The traditional observability analysis approaches are extended to linear and nonlinear deterministic systems with memory. By extending the definition of observability to deterministic systems with memory, a new relationship between the cumulative measurement and the initial state is formulated. Then, according to the implicit function theorem, the traditional observability rank condition for systems with memory is provided.

(2) A modified derivative-free approach based on the generalized Polynomial Chaos expansion for linear and nonlinear stochastic systems with memory is proposed, significantly reducing the computational cost and resolving the stochasticity of the system. Because the definition of observability is extended to stochastic systems with memory, it is necessary to represent this new relationship through a surrogate model. Thus, a different mapping between the stochastic input variable and standard normally distributed variable is formulated, resulting in a more appropriate surrogate model. Based on this model, the observability rank condition can subsequently be provided and its equivalence with the traditional approaches proven mathematically.

(3) Based on [15], several different observability indices, such as the first contribution rate and interference binary value, are proposed to describe the observability degree of systems, including the observability of each system state and the effect of measurement noise on the system observability.

(4) Finally, the proposed method is applied to observability analysis of a flexible flapping wing model inspired by the hawkmoth. Based on the proposed observability indices, the optimal sensor placement on the bioinspired wing is discussed and compared with the natural configuration of campaniform sensilla on the wing of the hawkmoth.

The remainder of the work is organized as follows. Section 2 formulates the flexible wing flapping dynamics model and neural encoding measurement model. Section 3 extends the traditional observability analysis approaches to linear and nonlinear systems with memory and discusses the shortcomings of the traditional approaches. Section 4 presents a derivative-free observability analysis approach and the observability indices for the linear and nonlinear system with memory, followed by the equivalence between the traditional and proposed approaches. Section 5 applies the proposed approach to the Lorenz system with memory and the bioinspired flexible flapping wing system. Section 6 discusses the optimal sensor placement based on the observability analysis. Finally, our conclusions are provided in Section 7.

## 2. System Model

This paper mainly focuses on the observability analysis problem of the bioinspired flexible flapping wing model based on neural encoded strain measurements. In this section, inspired by the North American hawkmoth, *Manduca sexta*, a low-order model of flexible wing flapping dynamics is first derived, followed by a neural encoding model of strain measurement.

### 2.1. Flexible Wing Flapping Dynamics

**Figure 1:**
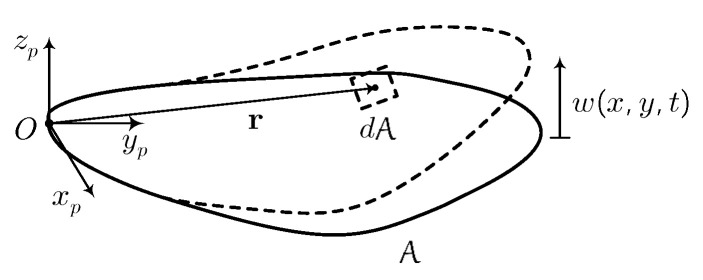
Wing Model in the Wing Body Frame.

Here, the flexible flapping wing is considered as a thin and flexible cantilevered plate A in a rotating coordinate system, as depicted in Figure 1. This coordinate system is called the wing body frame, where $x_{p}$ are the positive axis points from the leading edge to the trailing edge along the wing root, the positive axis $y_{p}$ is perpendicular to $x_{p}$ and points from root to tip in the wing plane, and the positive axis $z_{p}$ is perpendicular to the wing plane. The cantilevered plate is assumed to deform out-of-plane without in-plane stretching or extension.

Hence, the out-of-plane plate deformation w(x,y,t) is described by a finite number of orthonormal spatial modes, that is,

(1) $$w\left(x,y,t\right)=\sum_{i=0}^{n_{m}}\varphi_{i}\left(x,y\right)\eta_{i}\left(t\right)$$

where $\varphi_{i}\left(x,y\right)$ are the free-vibration mode shapes, $\eta_{i}\left(t\right)$ are the modal coordinates, and $n_{m}$ is the number of chosen modes used to represent the spatial deformation.

**Figure 2:**
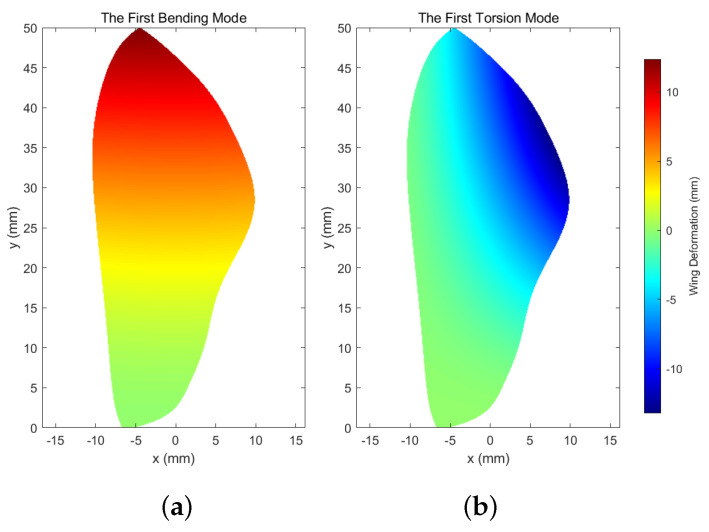
Prescribed wing mode shapes showing deformations in mm: (**a**) first bending mode and (**b**) first torsion mode.

Here, two main wing mode shapes, i.e., the first bending mode and the first torsion mode, are chosen to capture the principle wing deformations [8], as illustrated in Figure 2.

We define the unit vectors in each axis direction as $i_{p}$, $j_{p}$, and $k_{p}$, the position of a mass element dA on the plate in the wing body frame is

(2) $$r\left(x,y,t\right)=xi_{p}+yj_{p}+w\left(x,y,t\right)k_{p}$$

and the velocity of dA is provided by

(3) $$\begin{array}{cc} v & =v_{0}+\frac{\partial }{\partial t}r+\omega_{0}\times r\\ \; & =\left(U+Qw-Ry\right)i_{p}+\left(V+Rx-Pw\right)j_{p}+\left(W+\dot{w}+Py-Qx\right)k_{p} \end{array}$$

where $v_{0}=Ui_{p}+Vj_{p}+Wk_{p}$ is the velocity of the origin of wing body frame and $\omega_{0}=Pi_{p}+Qj_{p}+Rk_{p}$ is the angular velocity of wing body frame.

We define the mass density of the wing as ρ(x,y), the thickness as h(x,y), the kinetic energy as $T_{e}$, and the potential energy $U_{e}$ of the wing A as follows:

(4) $$\begin{array}{cc} T_{e}\left(t\right) & =\frac{1}{2}\int_{A}\rho \left(x,y\right)h\left(x,y\right)v\left(x,y,t\right)\cdot v\left(x,y,t\right)dA\\ U_{e}\left(t\right) & =\frac{1}{2}\int_{A}\frac{h{\left(x,y\right)}^{3}}{12}\Lambda {\left(x,y,t\right)}^{T}M\Lambda \left(x,y,t\right)dA \end{array}$$

where M is the matrix of the material constants and Λ is the strain vector:

(5) $$\Lambda ={\left[\begin{array}{ccc} \frac{\partial^{2}w}{\partial x^{2}} & \frac{\partial^{2}w}{\partial y^{2}} & \frac{2\partial^{2}w}{\partial x\partial y} \end{array}\right]}^{T}$$

Because the generalized coordinates describing the structural configuration are modal coordinates η, Lagrange’s equation is formulated as

(6) $$\frac{d}{dt}\left(\frac{\partial T_{e}}{\partial {\dot{\eta }}_{i}}\right)-\frac{\partial T_{e}}{\partial \eta_{i}}+\frac{\partial U_{e}}{\partial \eta_{i}}=Q_{i},\;i=1,\cdots ,n_{m}$$

where $Q_{i}$ are the exogenous non-conservative generalized forces.

Substituting Equations (1), (3), and (4) into Lagrange’s equation, the equations of motion for the flexible wing in the wing body frame is

(7) $$\ddot{\eta }+\Omega \eta +M_{a}\left(\left[\begin{array}{c} \dot{W}\\ \dot{P}\\ \dot{Q} \end{array}\right]-C_{a}\right)=Q$$

where the modal coordinate vector is $\eta ={\left[\eta_{1}\;\eta_{2}\right]}^{T}$, the total aerodynamic force is $Q={\left[Q_{1}\;Q_{2}\right]}^{T}$, and the stiff matrix Ω, applied acceleration mass matrix $M_{a}$, and Coriolis force $C_{a}$ are provided by Equations (8), (9), and (10), respectively:

(8) $$\Omega =\left[\begin{array}{cc} \omega_{1}^{2}-P^{2}-Q^{2} & 0\\ 0 & \omega_{2}^{2}-P^{2}-Q^{2} \end{array}\right]$$

where $\omega_{i}=2\pi f_{i}$ and $f_{i}$ denote the corresponding mode frequency of the *i*th mode shape.

(9) $$M_{a}=\int_{A}\left[\begin{array}{ccc} \rho h\varphi_{1} & \rho h\varphi_{1}y & -\rho h\varphi_{1}x\\ \rho h\varphi_{2} & \rho h\varphi_{2}y & -\rho h\varphi_{2}x \end{array}\right]dA$$

(10) $$C_{a}=\left[\begin{array}{c} QU-PV\\ -QR\\ PR \end{array}\right]$$

Let the position of the feathering rotation axis relative to the origin of the wing body frame be $r_{r}=x_{r}i_{p}+0j_{p}+0k_{p}$; then, the velocity and acceleration of wing body frame are computed by

(11) $$\begin{array}{cc} v_{o} & =-\omega_{0}\times r_{r}\\ {\dot{v}}_{o} & =-{\dot{\omega }}_{0}\times r_{r}-\omega_{0}\times \left(\omega_{0}\times r_{r}\right) \end{array}$$

Substituting Equation (11) into (7), the motion equation can be rewritten as

(12) $$\ddot{\eta }+\Omega \eta +M_{a}\left[\begin{array}{c} x_{r}\left(\dot{Q}-2PR\right)\\ \dot{P}+QR\\ \dot{Q}-PR \end{array}\right]=Q$$

The aerodynamics force Q is modeled as the aerodynamics model provided in [8], that is,

(13) $$Q=Q_{t0}+Q_{a0}+Q_{t\eta }\eta +Q_{a\dot{\eta }}\dot{\eta }+Q_{a\ddot{\eta }}\ddot{\eta }$$

where $Q_{t0}$ and $Q_{t\eta }$ denote the translational generalized force vector and translational force stiffness matrix, respectively, and $Q_{a0}$, $Q_{a\dot{\eta }}$, and $Q_{a\ddot{\eta }}$ represent the added mass force vector, added mass damping matrix, and added mass acceleration matrix, respectively. The detailed equations and corresponding derivations are omitted because the aerodynamics model is not the focus of this work.

Let the state vector be $x={\left[\eta \;\dot{\eta }\;P\;Q\;R\right]}^{T}$ and the control input vector be $u={\left[\dot{P}\;\dot{Q}\;\dot{R}\right]}^{T}$; then, the flexible wing flapping dynamics model is formulated by substituting Equation (13) into (12):

(14) $$\dot{x}=\left[\begin{array}{c} \dot{\eta }\\ {\left(I-Q_{a\ddot{\eta }}\right)}^{-1}\left[Q_{a\dot{\eta }}\dot{\eta }-\left(\Omega -Q_{t\eta }\right)\eta +Q_{t0}+Q_{a0}+M_{a_{1}}x_{r}\left(2PR-\dot{Q}\right)+M_{a_{2}}\left(-QR-\dot{P}\right)+M_{a_{3}}\left(PR-\dot{Q}\right)\right]\\ \dot{P}\\ \dot{Q}\\ \dot{R} \end{array}\right]$$

**Figure 3:**
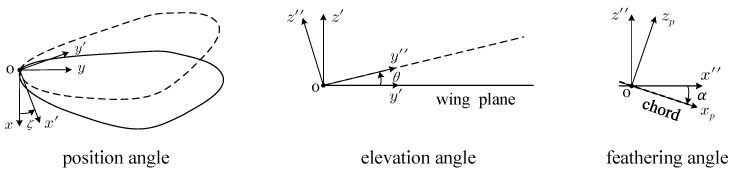
Wing model in a rotating reference frame.

Define the feathering angle as α, the elevation angle as θ, and the position angle as ζ, as shown in Figure 3; these three Euler angles represent the motion of the wing body frame with respect to the inertial frame.

Assuming that the 3-1-2 rotation sequence is used, $\omega_{0}$ and the Euler angles have the following relationship:

(15) $$\omega_{0}=\left[\begin{array}{ccc} cos\alpha & 0 & -sin\alpha cos\theta \\ 0 & 1 & sin\theta \\ sin\alpha & 0 & cos\alpha cos\theta \end{array}\right]\left[\begin{array}{c} \dot{\theta }\\ \dot{\alpha }\\ \dot{\zeta } \end{array}\right]$$

where $\dot{\theta }$, $\dot{\alpha }$, and $\dot{\zeta }$ denote the feathering, elevation, and position angular velocity, respectively. The control input u can be computed by taking the derivative of Equation (15) with respect to time *t*.

### 2.2. Neural Encoding Measurement Model

Assuming that a strain sensor on the wing plane is located at $r_{s}=x_{s}\vec{i_{p}}+y_{s}\vec{j_{p}}+z_{s}\vec{k_{p}}$, the strain measurement $\epsilon \left(x_{s},y_{s},t\right)={\left[\epsilon_{xx}\left(x_{s},y_{s},t\right)\;\epsilon_{yy}\left(x_{s},y_{s},t\right)\;\epsilon_{zz}\left(x_{s},y_{s},t\right)\right]}^{T}$ is provided by

(16) $$\epsilon \left(x_{s},y_{s},t\right)=-\sum_{i=1}^{n_{m}}z_{s}\left[\begin{array}{c} \frac{\partial^{2}\varphi_{i}\left(x_{s},y_{s}\right)}{\partial X^{2}}\\ \frac{\partial^{2}\varphi_{i}\left(x_{s},y_{s}\right)}{\partial y^{2}}\\ \frac{2\partial^{2}\varphi_{i}\left(x_{s},y_{s}\right)}{\partial x\partial y} \end{array}\right]\eta_{i}\left(t\right)$$

where $\epsilon_{xx}$, $\epsilon_{yy}$, and $\epsilon_{xy}$ mean the chord-wise bending strain, span-wise bending strain, and shear strain, respectively. Because the assumed structural modes do not have chord-wise bending, $\epsilon_{xx}$ is effectively zero, and only $\epsilon_{yy}$ and $\epsilon_{xy}$ are assumed to be measured by the sensors on the wing.

To determine the rotation rates, flying insects such as the hawkmoth *Manduca sexta* deliver the strain data from the campaniform sensilla on the wing to the central nervous system via sensory neurons, where the strain data are converted into action potentials (voltage spikes). This transformation process is called neural encoding. Inspired by this neural encoding mechanism, we constructed a neural encoding measurement model instead of using unprocessed strain measurements (16).

Several experiments have verified that the neural encoding model of hawkmoth wings is based on the two main functions [6,17], namely, the spike-triggered average (STA) and a nonlinear activation (NLA) functions.

The STA function is a measure that relates continuous strain stimulus to the spike, which represents the average stimuli taken during spike occurrences. This is approximated as an exponentially decaying sinusoidal function with a delay *a*

(17) $$STA\left(t\right)=cos\left(2\pi f_{STA}\left(-t+a\right)\right)exp\left(\frac{-{\left(-t+a\right)}^{2}}{b^{2}}\right)$$

where *b* is the width and $f_{STA}$ is the STA frequency.

The firing rate of a neuron $\varkappa (x_{s},y_{s},t)$ can be estimated by convolution of the strain stimulus and the STA:

(18) $$\varkappa \left(x_{s},y_{s},t\right)=\frac{1}{C_{\varkappa }}\int_{0}^{t_{M}}\epsilon \left(x_{s},y_{s},t-\tau \right)STA\left(\tau \right)d\tau$$

where $C_{\varkappa }$ is a normalization constant and $t_{M}$ is the memory length.

Furthermore, a saturation function is added to properly reflect the neuron’s nonlinear behaviour. Hence, by substituting $\varkappa (x_{s},y_{s},t)$ into the STA function, the probability of firing $P_{fire}\left(x_{s},y_{s},t\right)$, which implies the probability of firing an action potential at the coordinate $\left(x_{s},y_{s}\right)$ on the wing, is provided by

(19) $$\begin{array}{cc} P_{fire}\left(x_{s},y_{s},t\right) & =NLA(\varkappa \left(x_{s},y_{s},t\right))\\ \; & =\frac{1}{1+exp(-c\left(\varkappa \left(x_{s},y_{s},t\right)-d\right))} \end{array}$$

where *c* is the slope, *d* represents the half-maximum position of the NLA function, $P_{fire}\left(x_{s},y_{s},t\right)$ is regarded as the neural encoding strain measurement, and the neural encoding measurement model is provided by Equation (19).

## 3. Traditional Observability Analysis for Deterministic System with Memory

In this section, the traditional deterministic observability analysis methods are extended to both linear and nonlinear systems with memory; the limitations of the existing methods are then discussed.

### 3.1. Linear System with Memory

Consider the following general linear discrete-time time-invariant system with memory:

(20) $$\begin{array}{cc} x_{k+1} & =Ax_{k}+Bu_{k}\\ y_{k} & =\sum_{\tau =0}^{N}C_{\tau }x_{k-\tau } \end{array}$$

where $x_{k}\in R^{n\times 1}$, $u_{k}\in R^{n_{u}\times 1}$, and $y_{k}\in R^{m\times 1}$ are the state, control input, and measurement vector at time *k*, respectively. Here, *N* is the memory length, which implies that $y_{k}$ explicitly hinges on the information of states from time (k−N) to time *k* and that the measurement information can be obtained if and only if k≥N.

Hence, the definition of observability can be extended to the discrete-time dynamics system with memory (20) as follows:

**Definition** **1.** *The system is (locally) observable over the interval $\left[k_{1},k_{2}\right]$ if the initial state $x_{k_{1}-N}$ can be uniquely determined from $y_{k}$, $k\in [k_{1},k_{2}]$*.

The observability rank condition is determined by the following theorem:

**Theorem** **1.** *System* (20) *is (locally) observable if and only if the observability matrix*

(21) $$O=\left[\begin{array}{c} \bar{C}\\ \bar{C}A\\ \vdots \\ \bar{C}A^{n-1} \end{array}\right]$$

*is a full column matrix where $\bar{C}$ is defined as*

(22) $$\bar{C}=\sum_{\tau =0}^{N}C_{\tau }A^{N-\tau }$$

**Proof** **of Theorem 1.** Define the cumulative measurement vector $Y_{k}\in R^{mn\times 1}$ as

(23) $$Y_{k}={\left[\begin{array}{cccc} y_{k} & y_{k+1} & \cdots & y_{k+n-1} \end{array}\right]}^{T}$$

Substituting Equation (20) into Equation (23), the relationship between the arbitrary initial state $x_{k-N}$ and its corresponding measurements $Y_{k}$ is provided as

(24) $$Y_{k}=\left[\begin{array}{c} \bar{C}\\ \bar{C}A\\ \vdots \\ \bar{C}A^{n-1} \end{array}\right]x_{k-N}+D\left[\begin{array}{c} u_{k-1}\\ u_{k-2}\\ \vdots \\ u_{k-N} \end{array}\right]$$

where the (i,j) element of the feedthrough matrix $D\in R^{N\times (n-1)}$ is

(25) $$D\left(i,j\right)=\sum_{\tau =1}^{i}C_{\tau -1}A^{N+j-1-\tau }B$$

According to the implicit function theorem [18], the initial state $x_{k-N}$ can be uniquely determined from measurements $Y_{k}$ if and only if the Jacobian matrix

(26) $$O=\frac{\partial Y_{k}}{\partial x_{k-N}}$$

is a full column matrix.    □

The Gramian observability method provides an equivalent observability condition: the system (20) is (locally) observable if and only if the observability Gramian matrix

(27) $$W_{o_{k}}=\sum_{\tau =0}^{k}{\left(A^{T}\right)}^{\tau }{\bar{C}}^{T}\bar{C}A^{\tau }$$

is nonsingular.

These observability rank conditions make the observability analysis of a linear system simple and efficient. However, observability analysis become a significant challenge with regard to nonlinear systems and even to linear time-varying systems.

### 3.2. Nonlinear System with Memory

Now, consider a general continuous-time nonlinear system with memory

(28a) $$\begin{array}{cc} \dot{x}\left(t\right) & =f(x(t),u(t)) \end{array}$$

(28b) $$\begin{array}{cc} y(t) & =h(x\left(t\right),x\left(t-d_{1}\right),\cdots ,x\left(t-d_{N}\right)) \end{array}$$

where $x\left(t\right)\in R^{n\times 1}$, $u\left(t\right)\in R^{n_{u}\times 1}$ and $y\left(t\right)\in R^{m\times 1}$ are the state, control input, and measurement vector at time *t*(t≥N), respectively, $d_{i}=iD_{t}$ and $D_{t}$ is regarded as the time step, and f and h are vector-valued functions.

The definition of observability of the continuous-time dynamic system with memory (28) is then as follows.

**Definition** **2.** *The system is (locally) observable over the interval $\left[t_{1},t_{2}\right]$ if the initial state $x(t_{1}-d_{N})$ can be uniquely determined from y(t), $t\in [t_{1},t_{2}]$*.

According to Equation (28a), there obviously exists a function between $x\left(t-\tau \right),\tau =d_{0},d_{1},\cdots ,d_{N}$ and $x(t-d_{N})$; hence, the measurement (28b) can be rewritten as

(29) $$y\left(t\right)=\bar{h}\left(x\left(t-d_{N}\right),\bar{u}\left(t\right)\right)$$

where $\bar{u}\left(t\right)$ is a combination of known control inputs u from time $\left(t-d_{N}\right)$ to time *t*, denoted as $\bar{u}\left(t\right)=\left[u\left(t-d_{N}\right)\;u\left(t-d_{N-1}\right)\;\cdots \;u\left(t\right)\right]$. For simplicity of notation, we write $\bar{h}\left(x\left(t-d_{N}\right)\right)$ instead of Equation (29).

In this case, the observability rank condition is declared by the following theorem.

**Theorem** **2.** *System* (28) *is (locally) observable if and only if the observability matrix*

(30) $$O\left(t\right)={\left[\begin{array}{cccc} \frac{\partial L_{f}^{0}\bar{h}\left(x\left(t-d_{N}\right)\right)}{\partial x(t-d_{N})} & \frac{\partial L_{f}^{1}\bar{h}\left(x\left(t-d_{N}\right)\right)}{\partial x(t-d_{N})} & \cdots & \frac{\partial L_{f}^{n-1}\bar{h}\left(x\left(t-d_{N}\right)\right)}{\partial x(t-d_{N})} \end{array}\right]}^{T}$$

*is a full column matrix in which the Lie derivative is defined by*

(31) $$\begin{array}{cc} L_{f}^{0}\bar{h}\left(x\left(t-d_{N}\right)\right) & =\bar{h}\left(x\left(t-d_{N}\right)\right)\\ L_{f}^{k}\bar{h}\left(x\left(t-d_{N}\right)\right) & =\frac{\partial L_{f}^{k-1}\bar{h}\left(x\left(t-d_{N}\right)\right)}{\partial x(t-d_{N})}f\left(x\left(t-d_{N}\right)\right) \end{array}$$

**Proof** **of Theorem 2.** Define the cumulative measurement vector $Y\left(t\right)\in R^{mn\times 1}$ as

(32) $$Y\left(t\right)={\left[\begin{array}{cccc} y(t) & \dot{y}\left(t\right) & \cdots & y^{\left(n-1\right)}\left(t\right) \end{array}\right]}^{T}$$

According to the implicit function theorem [18], the initial state $x(t-d_{N})$ can be uniquely determined from measurements Y(t) if and only if

(33) $$rank(\frac{dY(t)}{dx(t-d_{N})})=n$$

With the chain rule, it is simple to obtain

(34) $${\bar{y}}^{\left(k\right)}\left(t\right)=L_{f}^{k}\bar{h}\left(x\left(t-d_{N}\right)\right)$$

Hence, the system is locally observable if condition (30) holds.    □

This differential geometric method provides a derivative-based analytical observability analysis approach for nonlinear systems called Lie derivative-based observability analysis. However, it usually encounters computational difficulties when solving the higher-order Lie derivatives of complex nonlinear systems, which makes it impracticable in real applications.

In analytically intractable cases [13], the most widely-used approach is the empirical observability Gramian approach [12], which provides a numerical way to approximate the traditional observability Gramian (27) by adding small perturbations in each initial state and comparing the output for each perturbation.

Define $y^{+i}$ as the output with the *i*th nominal initial state $x_{0,i}$ affected by a positive disturbance ϵ and let $y^{-i}$ be the output with $x_{0,i}$ affected by a negative disturbance −ϵ. The change in the measurement caused by perturbations in each initial state is denoted as $\Delta y_{i}=y^{+i}-y^{-i}$, and the empirical observability Gramian can be computed by

(35) $${\tilde{W}}_{o}\left(t\right)=\frac{1}{4\epsilon^{2}}\int_{0}^{t}\left[\begin{array}{c} \Delta y_{1}^{T}\\ \Delta y_{2}^{T}\\ \vdots \\ \Delta y_{r}^{T} \end{array}\right]\left[\begin{array}{cccc} \Delta y_{1} & \Delta y_{2} & \cdots & \Delta y_{r} \end{array}\right]d\tau$$

where *r* is the number of states of interest, which implies that the empirical observability Gramian is computed by simulating the system 2r times in total.

### 3.3. Limitations of Traditional Observability Analysis

Although these traditional methods are effective at providing a binary answer to the observability question, they have disadvantages which limit their practicality.

First, due to lack of a unified assessment metric, different systems correspond to different observability rank conditions, leading to confusion in the understanding and use of observability analysis. Second, because multiple derivative calculations are generally required in the analytical method for nonlinear systems, applying them to complex models, such as our bioinspired flexible wing model, is impractical. Furthermore, the traditional methods fail to precisely quantify each state’s observability. However, in observability analysis it is crucial to determine which states are observable and which are not. Finally, the existing methods are formulated in a deterministic framework, and hence, are not able to assess the observability of stochastic systems.

Therefore, a derivative-free observability analysis method is proposed here to overcome the above weaknesses. It is able to remarkably reduce the computational burden, analyze the observability degree of each state, and evaluate the effect of noise on observability.

## 4. gPC-Based Observability Analysis for Stochastic System with Memory

In this section, based on a brief review of the gPC expansion, the surrogate model of cumulative measurement is formulated, then the observability-coefficient matrix is provided for qualitative analysis of observability. Consequently, observability indices are proposed to quantify the observability of the system. Finally, the equivalence between the traditional and gPC-based methods for linear systems with memory is proven.

### 4.1. The Generalized Polynomial Chaos Expansion

The generalized Polynomial Chaos expansion is an efficient uncertainty propagation method that represents the stochastic output with a weighted sum of orthogonal polynomial chaos basis functions of random input variables.

Let *y* and $\xi =\left[\begin{array}{cccc} \xi_{1} & \xi_{2} & \cdots & \xi_{n} \end{array}\right]$ denote the output and random input variables, respectively, following a known probability distribution. The stochastic output can be expressed as

(36) $$y=\sum_{i=0}^{n_{p}}\gamma_{i}\phi_{i}\left(\xi \right)$$

where $\phi_{i}$ is a polynomial chaos basis function, $\gamma_{i}$ is the *i*th polynomial chaos coefficient, $n_{p}=\frac{(n+p)!}{n!p!}-1$, and *p* is the maximum order of the polynomial chaos basis functions.

The mean μ and variance $\sigma^{2}$ of output *y* can be directly obtained as

(37) $$\begin{array}{cc} \mu & =\gamma_{0}\\ \sigma^{2} & =\sum_{i=1}^{n_{p}}\gamma_{i}^{2} \end{array}$$

In general, higher orders yield higher accuracy. However, the number of unknown coefficients and the computational burden increase with the order. Many tests have proven that the increase in order has a negligible effect on the accuracy improvement when the order is larger than 2 [19]. Hence, the second order truncated gPC expansion is adopted in this paper, which is

(38) $$y=\gamma_{0}\phi_{0}+\sum_{i=1}^{n}\gamma_{i}\phi_{1}\left(\xi_{i}\right)+\sum_{i=1}^{n}\gamma_{i,i}\phi_{2}\left(\xi_{i}^{2}\right)$$

where $\phi_{0}$, $\phi_{1}\left(\xi_{i}\right)$, and $\phi_{2}\left(\xi_{i}^{2}\right)$ denote the zeroth-order, first-order, and second-order polynomial chaos bases, respectively, and $\gamma_{0}$, $\gamma_{i}$, and $\gamma_{i,i}$ represent the corresponding polynomial chaos coefficients. Note that only 2n+1 polynomial chaos coefficients need to be determined.

Assuming that the random variable $\xi_{i}$ follows a standard normal distribution, the corresponding polynomial chaos bases are as follows [19]:

(39) $$\begin{array}{cc} \; & \phi_{0}=1\\ \; & \phi_{1}\left(\xi \right)=\xi \\ \; & \phi_{2}\left(\xi \right)=\frac{\xi^{2}-1}{\sqrt{2}} \end{array}$$

### 4.2. Observability Rank Condition

Consider a general nonlinear stochastic system with memory (28), where $x=[x_{1}\;x_{2}\;\cdots \;x_{n}]$ and the *i*th state random variable $x_{i}$ follows the known distribution.

Distinct from [15], the definition of observability of stochastic systems with memory is extended as follows.

**Definition** **3.** *The system is (locally) observable over the interval $\left[t_{1},t_{2}\right]$ if the initial state $x(t_{1}-N)$ can be inferred from y(t), $t\in [t_{1},t_{2}]$ and its solution satisfies a 95% confidence interval*.

In order to resolve probabilistic uncertainty propagation in such a system, a surrogate model is formulated by the following steps to represent the above relationship between the random initial state $x(t-d_{N})$ and its corresponding cumulative measurement vector Y(t) (32).

First, we determine the mapping relationship between the *i*th element of the stochastic input variable $x(t-d_{N})$ and a random variable $\xi_{i}$ following a standard normal distribution, which is

(40) $$x_{i}\left(t-d_{N}\right)=F_{i}^{-1}\left(T_{i}\left(\xi_{i}\right)\right)$$

where $F_{i}$ is the cumulative probability function of $x_{i}\left(t-d_{N}\right)$, $F_{i}^{-1}$ is the inverse function of $F_{i}$, and $T_{i}$ denotes the cumulative probability function of $\xi_{i}$.

Next, collocations points (CPs) are selected to construct the surrogate model. CPs are a finite sample set of ξ, and the *i*th combination of CPs is expressed as $\xi_{i}=\left[\xi_{i,1}\;\xi_{i,2}\;\cdots \;\xi_{i,n}\right]$. The element values of the CPs are generated by the roots of one higher-order one-dimensional Hermite polynomial [19]. For example, three roots of the third order Hermite polynomial $\phi_{3}\left(\xi \right)=\frac{\xi^{3}-3\xi }{\sqrt{6}}$, which are $\left\{-\sqrt{3},0,\sqrt{3}\right\}$, are used to to comprise the CPs, as a second order polynomial chaos expansion is adopted in this paper. Because there are 2n+1 unknown polynomial chaos coefficients, 2n+1 independent combinations of CPs are chosen randomly from the $3^{n}$ possible combinations, which results in the matrix $\Xi \in R^{(2n+1)\times n}$, as follows:

(41) $$\Xi =\left[\begin{array}{cccc} \xi_{1,1} & \xi_{1,2} & \cdots & \xi_{1,n}\\ \xi_{2,1} & \xi_{2,2} & \cdots & \xi_{2,n}\\ \vdots & \vdots & \ddots & \vdots \\ \xi_{2n+1,1} & \xi_{2n+1,2} & \cdots & \xi_{2n+1,n} \end{array}\right]$$

which is the full rank where the value of the *i*th element of the *s*th CP $\xi_{s,i}$ is chosen randomly from $\left\{-\sqrt{3},0,\sqrt{3}\right\}$.

Then, 2n+1 samples of stochastic input variables are transformed from 2n+1 CPs based on Equation (40). Substituting them into Equation (32) in place of the original input variables $x(t-d_{N})$, the output matrix $Y\left(t\right)\in R^{(2n+1)\times mn}$ is obtained, where $Y\left(t\right)={\left[Y_{1}\left(t\right)\;Y_{2}\left(t\right)\;\cdots \;Y_{2n+1}\left(t\right)\right]}^{T}$ and $Y_{i}$ represents the measurement output from the *i*th sample.

Consequently, according to Equation (38), the surrogate model is provided by

(42) Y(t)=HΓ(t)

where the basis matrix of polynomial chaos bases $H\in R^{\left(2n+1)\times (2n+1\right)}$ is provided by Equation (43) and the coefficient matrix of polynomial chaos coefficients $\Gamma \left(t\right)\in R^{(2n+1)\times mn}$ is Equation (44); here, $\gamma_{0}^{l}$, $\gamma_{i}^{l}$, and $\gamma_{i,i}^{l}$ represent the polynomial chaos coefficients with respect to the *i*th state and *l*th measurement:

(43) $$H=\left[\begin{array}{ccccccccc} \phi_{0} & \phi_{1}\left(\xi_{1,1}\right) & \phi_{1}\left(\xi_{1,2}\right) & \cdots & \phi_{1}\left(\xi_{1,n}\right) & \phi_{2}\left(\xi_{1,1}^{2}\right) & \phi_{2}\left(\xi_{1,2}^{2}\right) & \cdots & \phi_{2}\left(\xi_{1,n}^{2}\right)\\ \phi_{0} & \phi_{1}\left(\xi_{2,1}\right) & \phi_{1}\left(\xi_{2,2}\right) & \cdots & \phi_{1}\left(\xi_{2,n}\right) & \phi_{2}\left(\xi_{2,1}^{2}\right) & \phi_{2}\left(\xi_{2,2}^{2}\right) & \cdots & \phi_{2}\left(\xi_{2,n}^{2}\right)\\ \vdots & \vdots & \vdots & \ddots & \vdots & \vdots & \vdots & \ddots & \vdots \\ \phi_{0} & \phi_{1}\left(\xi_{2n+1,1}\right) & \phi_{1}\left(\xi_{2n+1,2}\right) & \cdots & \phi_{1}\left(\xi_{2n+1,n}\right) & \phi_{2}\left(\xi_{2n+1,1}^{2}\right) & \phi_{2}\left(\xi_{2n+1,2}^{2}\right) & \cdots & \phi_{2}\left(\xi_{2n+1,n}^{2}\right) \end{array}\right]$$

(44) $$\Gamma \left(t\right)=\left[\begin{array}{cccc} \gamma_{0}^{1} & \gamma_{0}^{2} & \cdots & \gamma_{0}^{mn}\\ \gamma_{1}^{1} & \gamma_{1}^{2} & \cdots & \gamma_{1}^{mn}\\ \vdots & \vdots & \ddots & \vdots \\ \gamma_{n}^{1} & \gamma_{n}^{2} & \cdots & \gamma_{n}^{mn}\\ \gamma_{1,1}^{1} & \gamma_{1,1}^{2} & \cdots & \gamma_{1,1}^{mn}\\ \vdots & \vdots & \ddots & \vdots \\ \gamma_{n,n}^{1} & \gamma_{n,n}^{2} & \cdots & \gamma_{n,n}^{mn} \end{array}\right]$$

Because Ξ is full rank, it is obvious that H is nonsingular and its inverse matrix $H^{-1}$ exists. Hence, the unknown polynomial chaos coefficients can be obtained by

(45) $$\Gamma \left(t\right)=H^{-1}Y\left(t\right)$$

According to Equation (37) and the orthogonal property [20], the zero-order polynomial chaos coefficient $\gamma_{0}^{l}$ denotes the mean of the estimated value for the *l*th measurement and the first and second order polynomial chaos coefficients $\gamma_{i}^{l}$ and $\gamma_{i,i}^{l}$ represent the contribution of the *i*th state to the uncertainty of the *l*th measurement. Supposing that the contribution of the *i*th state to the uncertainty of the *l*th measurement is zero, the *l*th measurement is unaffected by the change in this state, which implies that the *i*th state cannot be uniquely determined from the *l*th measurement. In order to uniquely infer *n* states from the given measurements, *n* valid measurements, for which the contributions of the states to the uncertainties of the estimated values for these measurements are linearly independent, are necessary.

Therefore, we define the observability coefficient matrix $\Phi \left(t\right)\in R^{2n\times mn}$ as follows:

(46) $$\Phi \left(t\right)=\left[\begin{array}{cccc} \gamma_{1}^{1} & \gamma_{1}^{2} & \cdots & \gamma_{1}^{mn}\\ \vdots & \vdots & \ddots & \vdots \\ \gamma_{n}^{1} & \gamma_{n}^{2} & \cdots & \gamma_{n}^{mn}\\ \gamma_{1,1}^{1} & \gamma_{1,1}^{2} & \cdots & \gamma_{1,1}^{mn}\\ \vdots & \vdots & \ddots & \vdots \\ \gamma_{n,n}^{1} & \gamma_{n,n}^{2} & \cdots & \gamma_{n,n}^{mn} \end{array}\right]$$

and the observability rank condition is stated by the following theorem.

**Theorem** **3.** *System* (28) *is (locally) observable if and only if the observability coefficient matrix Φ(t) has n linearly independent column vectors and the ith and (n+i)th rows are not zero vectors*.

Note that by using a surrogate model to represent $Y_{k}$ in Equation (23), the proposed gPC-based observability analysis method can solve the observability problem of linear and nonlinear discrete-time systems with memory.

The detailed gPC-based observability analysis procedure is summarized in Algorithm 1.
**Algorithm 1** gPC-based Observability Analysis Procedure1:Determine the mapping between *i*th random variable $x_{i}\left(t-d_{N}\right)$ and a normal variable $\xi_{i}$ via Equation (40);2:Select CPs (41) based on the linear independence method and substitute them into basis matrix (43);3:Transform all CPs into the samples of stochastic input variables based on the mapping (40);4:Compute output matrix Y(t);5:Calculate the coefficient matrix (45) and the observability-coefficient matrix Φ(t);6:Analyze Observability according to the observability rank condition Theorem 3.

### 4.3. Degree of Observability

The observability rank condition can only draw a binary conclusion as to whether the system is observable. However, it is limited to quantifying the observability of the system. Hence, three more quantitative observability indices are proposed to further analyze the observability degree.

1. *Condition Number*: The condition number κ(Φ) is the ratio of the maximum singular value to the minimum singular value, which is utilized to show the numerical stability of the system states, and is provided by
  (47) $$\kappa \left(\Phi \right)=\frac{\sigma_{max}\left(\Phi \right)}{\sigma_{min}\left(\Phi \right)}$$
  where $\sigma_{max}\left(\Phi \right)$ and $\sigma_{min}\left(\Phi \right)$ are the maximal and minimal singular values of Φ, respectively.
  Suppose that the condition number is enormous or even goes to infinity, the observability-coefficient matrix Φ is ill-conditioned, and the system is weakly observable; this implies that certain states are hard to observe. However, if the condition number is close to one, Φ is well-conditioned and the system is brawny observable. Note that all system states are considered equally observable when the condition number equals one.
2. *Contribution Rate*: Two different equations for the contribution rate are provided in this paper, both of which are regarded as quantitative indices of each state’s observability. The first contribution rate, $\chi_{i}^{1}$, is defined as the contribution of the *i*th state to the uncertainty of all measurements. According to the surrogate model, it is provided by
  (48) $$\chi_{i}^{1}=\frac{\sum_{l=1}^{mn}{\left(\gamma_{i}^{l}\right)}^{2}+{\left(\gamma_{i,i}^{l}\right)}^{2}}{\sum_{i=1}^{n}\sum_{l=1}^{mn}{\left(\gamma_{i}^{l}\right)}^{2}+{\left(\gamma_{i,i}^{l}\right)}^{2}}$$
  which represents the influence of a specific state on the measurements. The *i*th state is brawnier observable when its first contribution rate $\chi_{i}^{1}$ is closer to one. Instead, the *i*th state is weakly observable as $\chi_{i}^{1}$ approaches zero.
  The second contribution rate, $\chi_{i}^{2}$, is the maximum contribution of the *i*th state to each measurement. We define the proportion of the contribution of the *i*th state to the variance of the *l*th measurement as $\chi_{i,l}^{2}$, that is,
  (49) $$\chi_{i,l}^{2}=\frac{{\left(\gamma_{i}^{l}\right)}^{2}+{\left(\gamma_{i,i}^{l}\right)}^{2}}{\sum_{i=1}^{n}{\left(\gamma_{i}^{l}\right)}^{2}+{\left(\gamma_{i,i}^{l}\right)}^{2}}$$
  where the numerator means the contribution of the *i*th state to the variance of the *l*th measurement and the denominator denotes the variance of the *l*th measurement.
  Hence, $\chi_{i}^{2}$ equals the maximum of $\left(\chi_{i,1}^{2},\chi_{i,2}^{2},\cdots ,\chi_{i,mn}^{2}\right)$. As in the first contribution rate, the larger the second contribution rate is, the more the state is brawnier observable.
3. *Interference Rate*: If the contribution of the *i*th state to the variance of the *l*th measurement, ${\left(\gamma_{i}^{l}\right)}^{2}+{\left(\gamma_{i,i}^{l}\right)}^{2}$, is smaller than the measurement noise variance $\sigma_{v}^{2}$, the changes in measurements contain too much environmental interference, and the initial state is difficult to distinguish from noisy measurements with a high confidence level.
  Therefore, we define the proportion of the measurement noise variance $\sigma_{v}^{2}$ to the variance of the *l*th measurement as the interference rate:
  (50) $$V_{l}=\frac{\sigma_{v}^{2}}{\sum_{i=1}^{n}{\left(\gamma_{i}^{l}\right)}^{2}+{\left(\gamma_{i,i}^{l}\right)}^{2}}$$
  If the interference rates $V_{1},V_{2},\cdots ,V_{mn}$ are larger than all the corresponding contribution of any states $\chi_{i,1}^{2},\chi_{i,2}^{2},\cdots ,\chi_{i,mn}^{2}$, the system is considered to be weakly observable due to noisy measurements.
  To simplification simulation, an equivalent index called the interference binary value Υ is used:
  (51) $$\Upsilon =\left\{\begin{array}{c} 0\;\max_{i}\min_{l}V_{l}/\chi_{i,l}^{2}\leq 1\\ 1\;otherwise \end{array}\right.$$
  When Υ equals zero, the measurement noise has negligible effect on observability analysis. Inversely, the states are hard to infer from noisy measurements when Υ equals one.

### 4.4. Equivalence between the Traditional and Proposed Approaches

To further clarify the rationality of the proposed method from another perspective, the connection between the traditional and proposed methods for a general linear system with memory (20) is demonstrated as a particular case.

For a general linear system with memory (20), the relationship between the random initial state $x_{k-N}$ and the cumulative measurement $Y_{k}$ at time *k* is provided by Equation (24). We define $\Delta x_{k-N}\in R^{n\times 1}$ and $\Delta Y_{k}\in R^{m\times 1}$ as the state and measurement uncertainty vectors, respectively; as the control input vectors u from time k−N to k−1 are determined at time *k*, the uncertainty propagation function is

(52) $$\Delta Y_{k}=O\Delta x_{k-N}$$

where O is provided by Equation (21).

Assuming that there exists an open neighborhood *U* of $x_{k-N}$ and that $x_{k-N}+\Delta x_{k-N}\in U$, we can find that $x_{k-N}$ and $x_{k-N}+\Delta x_{k-N}$ are distinguishable from their corresponding measurements if and only if the observability matrix O has full column ranking based on Theorem 4, which implies that system (20) is locally observable at time *k*.

**Theorem** **4.** *Consider a non-homogeneous linear system of equations as*

(53) Ax=b

*where $A\in R^{m\times n}$, $x\in R^{n\times 1}$, and $b\in R^{m\times 1}$*.

*Then, there exists a unique solution if and only if the rank of the coefficient matrix A is n*.

Taking *n* samples of stochastic input variables into consideration, the above equation can be rewritten as

(54) ΔY=ΔXO

where $\Delta Y={\left[\Delta Y_{k}^{1},\Delta Y_{k}^{2},\cdots ,\Delta Y_{k}^{n}\right]}^{T}$ and $\Delta X={\left[\Delta x_{k-N}^{1},\Delta x_{k-N}^{2},\cdots ,\Delta x_{k-N}^{n}\right]}^{T}$. $\Delta Y_{k}^{i}$, where $\Delta x_{k}^{i}$ represents the measurement and state uncertainty vector from *i*th samples at time *k*.

In the proposed approach, the surrogate model of the relationship between $x_{k-N}$ and $Y_{k}$ is expressed as

(55) $$Y_{k}=H\Gamma_{k}$$

Because the second-order polynomial chaos coefficients are zeros for a linear system, the surrogate model is simplified as

(56) $$Y_{k}=\bar{H}{\bar{\Gamma }}_{k}$$

where

(57) $$\bar{H}=\left[\begin{array}{ccccc} \phi_{0} & \phi_{1}\left(\xi_{1,1}\right) & \phi_{1}\left(\xi_{1,2}\right) & \cdots & \phi_{1}\left(\xi_{1,n}\right)\\ \phi_{0} & \phi_{1}\left(\xi_{2,1}\right) & \phi_{1}\left(\xi_{2,2}\right) & \cdots & \phi_{1}\left(\xi_{2,n}\right)\\ \vdots & \vdots & \vdots & \ddots & \vdots \\ \phi_{0} & \phi_{1}\left(\xi_{n+1,1}\right) & \phi_{1}\left(\xi_{n+1,2}\right) & \cdots & \phi_{1}\left(\xi_{n+1,n}\right) \end{array}\right]$$

(58) $${\bar{\Gamma }}_{k}=\left[\begin{array}{cccc} \gamma_{0}^{1} & \gamma_{0}^{2} & \cdots & \gamma_{0}^{mn}\\ \gamma_{1}^{1} & \gamma_{1}^{2} & \cdots & \gamma_{1}^{mn}\\ \vdots & \vdots & \ddots & \vdots \\ \gamma_{n}^{1} & \gamma_{n}^{2} & \cdots & \gamma_{n}^{mn} \end{array}\right]$$

As mentioned above, $\gamma_{0}^{l}$ is the mean of the *l*th measurement and $\gamma_{i}^{l}$ stands for the contribution of the *i*th state to the uncertainty of the *l*th measurement. Hence,

(59) $$\Delta Y=\Delta X\bar{\Phi }$$

where

(60) $$\bar{\Phi }=\left[\begin{array}{cccc} \gamma_{1}^{1} & \gamma_{1}^{2} & \cdots & \gamma_{1}^{mn}\\ \vdots & \vdots & \ddots & \vdots \\ \gamma_{n}^{1} & \gamma_{n}^{2} & \cdots & \gamma_{n}^{mn} \end{array}\right]$$

System (20) is locally observable if and only if $rank(\bar{\Phi })=n$.

The same observability rank condition for linear systems with memory are derived from the traditional and proposed method. This convincingly proves that gPC-based observability analysis is equivalent to the traditional method.

## 5. Simulations

In this section, the observability of a Lorenz system with memory is first analyzed by the traditional and proposed methods to validate the feasibility of gPC-based observability analysis. Then, we apply the proposed method to observability analysis of a bioinspired flapping wing system based on the hawkmoth, *Manduca sexta*.

### 5.1. Lorenz System with Memory

Consider the following Lorenz system with memory:

(61) $$\begin{array}{cc} \; & \left\{\begin{array}{c} {\dot{x}}_{1}\left(t\right)=-10x_{1}\left(t\right)+10x_{2}\left(t\right)\\ {\dot{x}}_{2}\left(t\right)=28x_{1}\left(t\right)+x_{1}\left(t\right)x_{3}\left(t\right)-x_{2}\left(t\right)\\ {\dot{x}}_{3}\left(t\right)=x_{1}\left(t\right)x_{2}\left(t\right)-\frac{8}{3}x_{3}\left(t\right) \end{array}\right.\\ \; & \left\{\begin{array}{c} y_{1}\left(t\right)=x_{1}\left(t\right)+\frac{1}{2}x_{1}\left(t-d_{1}\right)+\frac{1}{4}x_{1}\left(t-d_{2}\right)\\ y_{2}\left(t\right)=x_{2}\left(t\right)+\frac{1}{2}x_{2}\left(t-d_{1}\right)+\frac{1}{4}x_{2}\left(t-d_{2}\right) \end{array}\right. \end{array}$$

with starting time $t_{0}=0s$, final time $t_{f}=10s$, time step dt=0.01s, and initial state $x_{1}\left(0\right)=x_{2}\left(0\right)=x_{3}\left(0\right)=1$.

#### 5.1.1. Lie Derivative-Based Observability Analysis

**Figure 4:**
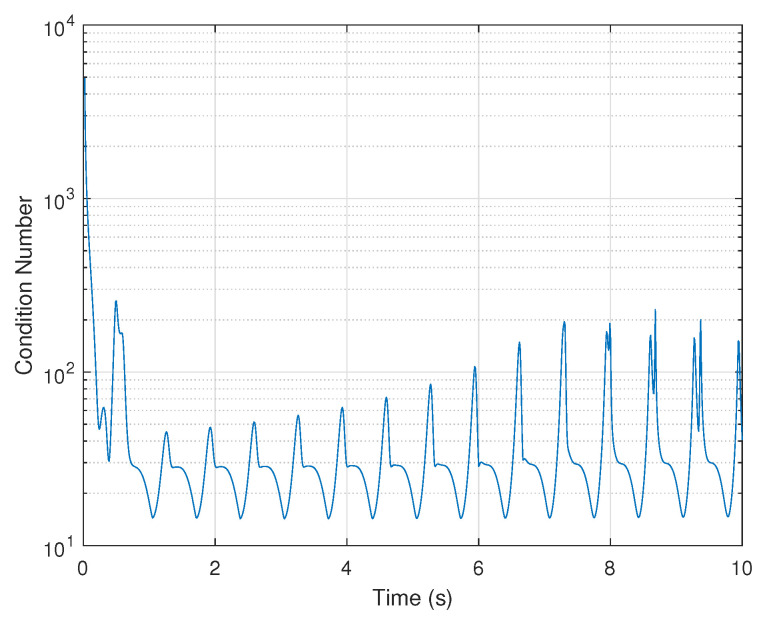
Lie Derivative-based observability analysis: condition number of observability matrix.

The observability analysis is first performed by using the Lie derivative-based approach. The observability matrix O(t) is calculated by Equation (32), and its rank is shown in Table 1. In addition, the condition number of O(t) is shown in Figure 4 to measure the observability degree.

It can be seen that the system is observable, as the observability matrix is full rank and the value of the condition number changes drastically and reaches large values at intervals, revealing that the system is weakly observable. However, this approach can neither quantify the degree of observability for each state nor account for the effect of the observation noise, thereby reducing its reliability in practice.

#### 5.1.2. Empirical Observability Gramian Analysis

**Figure 5:**
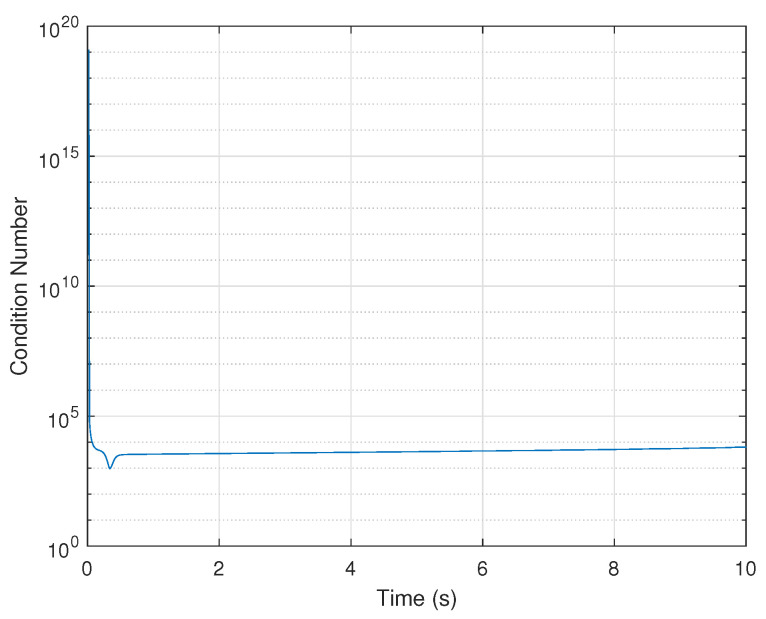
Empirical observability Gramian analysis: condition number of observability matrix.

Empirical observability Gramian is the most widely used method in practical applications due to its avoidance of the need to calculate the complicated Lie derivatives of nonlinear systems. The rank of the observability matrix ${\tilde{W}}_{o}\left(t\right)$ computed by Equation (35) is shown in Table 1, and the condition number is present in Figure 5.

The same conclusion that the system is weakly observable is drawn via empirical observability Gramian analysis, as the observability matrix maintains full rank while the condition numbers are far from one. However, the condition number is more stable than that computed by the Lie derivative-based approach, most likely due to the inaccuracy of the approximation.

#### 5.1.3. gPC-Based Observability Analysis

Here, the proposed observability analysis approach is utilized. The observability coefficient matrix is computed through Equation (44). In the observability coefficient matrix, the first order polynomial chaos coefficients are larger and more significant than the second order polynomial chaos coefficients [16]; thus, we name the first *n* rows of the observability coefficient matrix as the first order observability coefficient matrix. The ranks of these two observability matrices are shown in Table 1. It can be seen that both of the observability coefficient matrices are full rank at all times, meaning that the system is observable.

**Figure 6:**
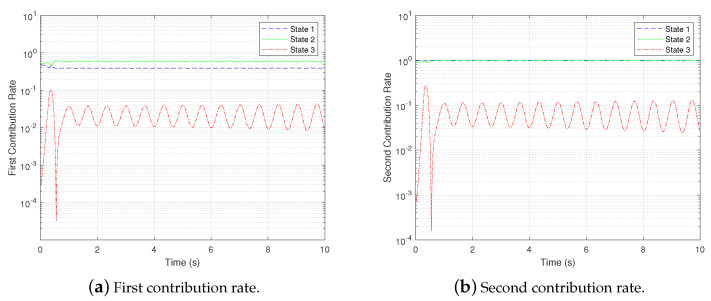
gPC-based observability Gramian analysis: contribution rates.

Then, the first and second contribution rates of each state are shown in Figure 6.

It is evident from Figure 6 that the contribution rate of the third state is much lower than that of the first two states and varies periodically with time, which implies that the third state is weakly observable while the others are brawny observable.

**Figure 7:**
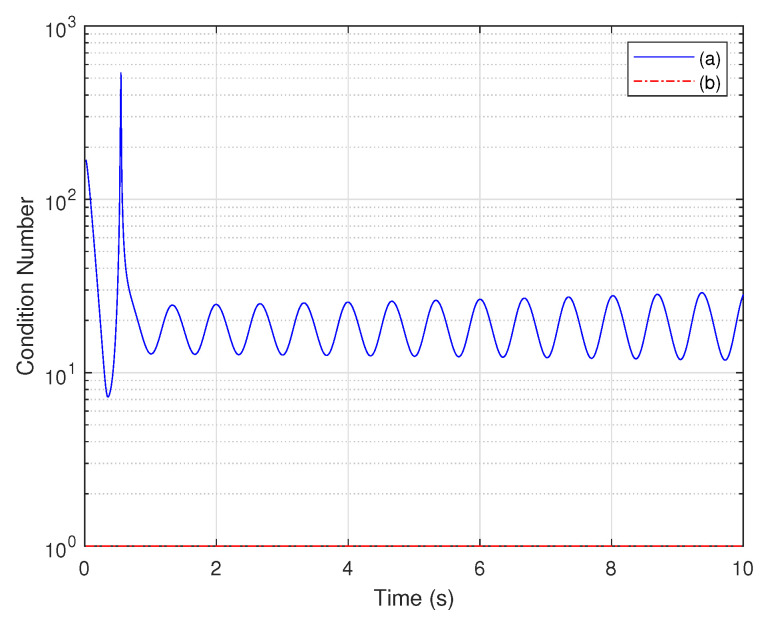
gPC-based observability Gramian analysis: condition number of observability matrix: (a) first-order coefficient matrix and (b) active coefficient matrix.

This conclusion can be proven by the condition number. The condition number of the first order coefficient matrix is displayed in Figure 7a. It can be seen that it has enormous values and a similar change to the contribution rate of the third state. This is because the weak observability of the third state has a negative impact on the observability of the whole system, thereby resulting in an ill-conditioned matrix.

The coefficients of the states with brawny observability extracted from the first-order coefficient matrix constitute the active coefficient matrix. Its condition number is presented in Figure 7b, showing that the condition number of the active coefficient matrix is very close to one. This implies that the active coefficient matrix is well-conditioned and the first two states are brawny observable. Therefore, it can be concluded that the condition number is strongly affected by the weak observability of any state. When the condition number has large values, at least one state of the system is weakly observable.

**Figure 8:**
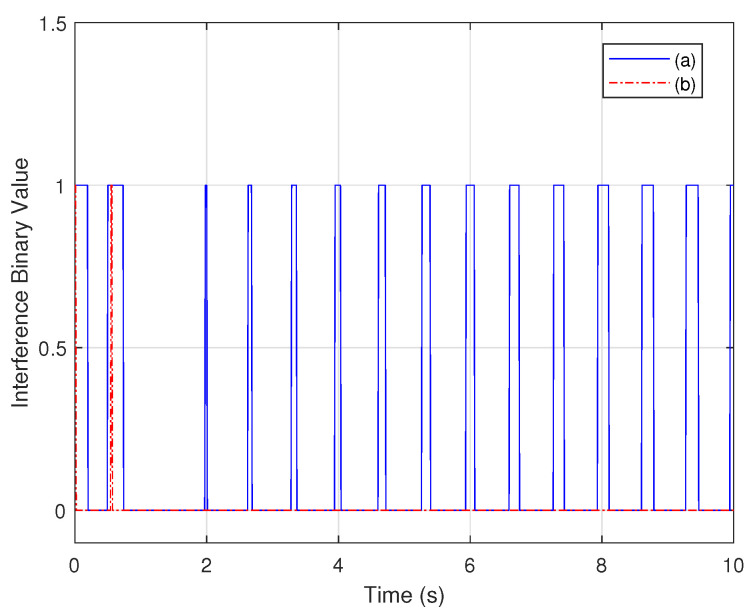
gPC-based observability Gramian analysis interference binary value: (a) large noise variance and (b) small noise variance.

In addition, the effect of the measurement noise on the observability is shown in Figure 8. Two different noise variances are assumed, $1\times 10^{-9}$ and $1\times 10^{-11}$. When the noise variance becomes larger, more interference binary values within the simulation time equal one, which implies that the observability of the system is weaker.

Finally, the computation complexity of the three approaches is compared by computation time in Table 2. The computation time was computed using MATLAB R2021b on a 2.70 GHz Intel(R) Core(TM) i7 processor with 8 GB RAM.

Though Lie derivative-based observability analysis has the shortest time cost in the case where the derivation is completed, it is limited for systems with high nonlinearity in actual application because of the complicated derivative calculation. The proposed method effectively reduces the computational cost compared to empirical observability Gramian analysis.

### 5.2. Bioinspired Flexible Flapping Wing System

Considering the system model as Equation (14) and the measurement model as Equation (19), the proposed observability analysis approach is used to determine whether the neural encoding strain measurements are sufficient to reconstruct the wing rotation rates (*P*, *Q*, *R*). The observability analysis plays a vital role in the optimal placement of sensors on the wing in practice, which is further discussed in the next section.

The simulation of the full bioinspired flexible flapping wing model requires several steps. First, the wing-beat period and the total simulation time are assumed to be $T_{beat}$ and $2T_{beat}$, respectively. The time step is set as $T_{beat}/50$. The first period is used to fill the memory, and the observability analysis is based on the measurements at the second period.

Second, the control input sequence $u\left(t\right)={\left[\dot{P}\left(t\right)\;\dot{Q}\left(t\right)\;\dot{R}\left(t\right)\right]}^{T}$ is generated by derivation of Equation (15) with respect to time *t*. According to [21,22], the Euler angles are provided by

(62) $$\begin{array}{cc} \theta (t) & =0\\ \alpha (t) & =\frac{\pi }{2}-A_{\alpha }tanh\left(\frac{\pi }{2}sin\left(\frac{2\pi t}{T_{beat}}\right)\right)\\ \zeta (t) & =-A_{\zeta }cos\left(\frac{2\pi t}{T_{beat}}\right) \end{array}$$

where $A_{\zeta }$ is the position angle amplitude and $A_{\alpha }$ is the feathering angle amplitude.

Then, the stiff matrix Ω, applied acceleration mass matrix $M_{a}$, and aerodynamics force Q are computed using the wing structural mode shapes $\varphi_{i}\left(x,y\right)$, their corresponding $f_{i}$, the mass density ρ(x,y), and the thickness h(x,y). We assume that the mass density of the wing is constant and model the thickness as an exponential decrease from root to tip and from the leading edge (LE) to the trailing edge (TE) [23], that is,

(63) $$h\left(x,y\right)=\frac{1}{2}\left(t_{LE}e^{-a_{LE}\frac{y}{c_{wing}}}+t_{RO}e^{-a_{RO}\frac{x-x_{LE}\left(y\right)}{d_{wing}}}\right)$$

where $t_{LE}$ and $t_{RO}$ are the leading edge and the root thickness, respectively, $c_{wing}$ and $d_{wing}$ are the chord and spanwise length, respectively, $a_{LE}$ and $a_{RO}$ are the respective decay rates along the two directions, $x_{LE}\left(y_{0}\right)$ means the x-ordinate of the intersection point at the leading edge, and $y=y_{0}$.

Finally, bysubstituting these pre-processing data and the detailed parameters listed in Table 3 into Equations (14) and (19), the full bioinspired flexible flapping wing model is constructed and the observability indices are calculated using the gPC-based observability analysis approach.

To account for the observability changes on different locations of different sensors, the wing’s surface is meshed into a 50×22 grid. One shear sensor and one bending sensor are placed at each point of intersection on the wing. Here, the first contribution rate is adopted to describe the observability degree of each state, while the minimum contribution rate provides a measure of the weakest observable state.

**Figure 9:**
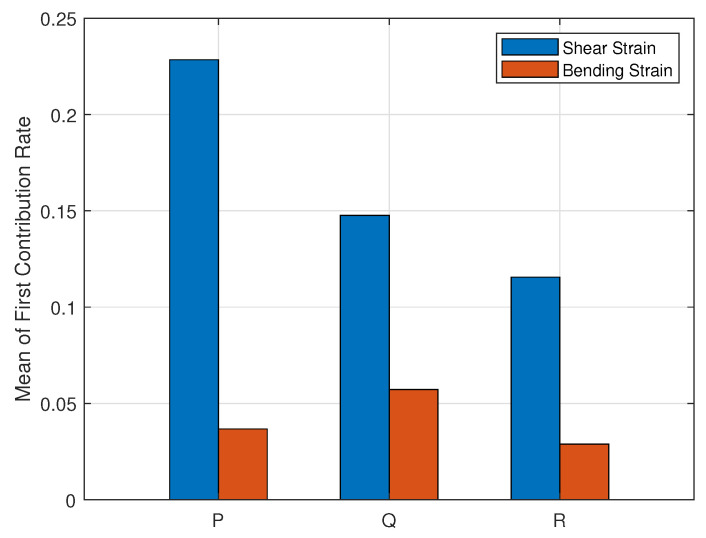
Averaged first contribution rates of wing rotation rates for all sensors.

The averaged contribution rate of the three wing rotation rates for all sensors is shown in Figure 9.

The above illustrates that R is the weakest observable state when only shear or bending strain measurements are obtained, while P and Q are brawnier observable. In addition, shear strain measurements result in brawnier observability of the whole wing model.

On the other hand, bending strain measurements provide the smaller condition number, with a mean value of 52.5629, while the averaged condition number of the shear strain measurements is 87.049, which implies that the observability of all rotation rates are more balanced when using bending strain measurements.

**Figure 10:**
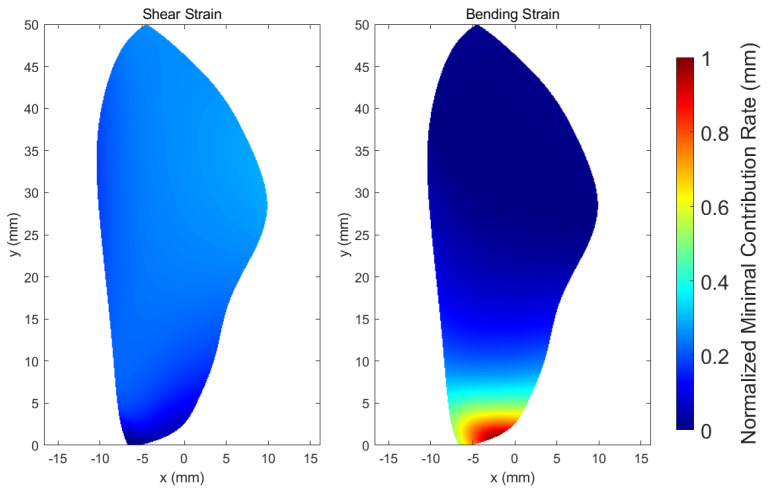
Averaged minimum contribution rate on the wing normalized via the max–min normalization method.

Then, the averaged minimum contribution rate for each sensor location and type within the simulation time is obtained and normalized by the max-min normalization method, depicted in Figure 10.

This indicates that the rotation rates can be accurately estimated when bending strain sensors are concentrated in the wing root and shear strain sensors are placed in the upper part of the trailing edge, as because the bending strain at the wing root and shear strain at the upper part of the trailing edge are the largest and contain more information about the rotation rates. In addition, bending strain sensors located in the wing root provide more measurements to encode the rotation rate compared to the shear strain sensors at their best location.

**Figure 11:**
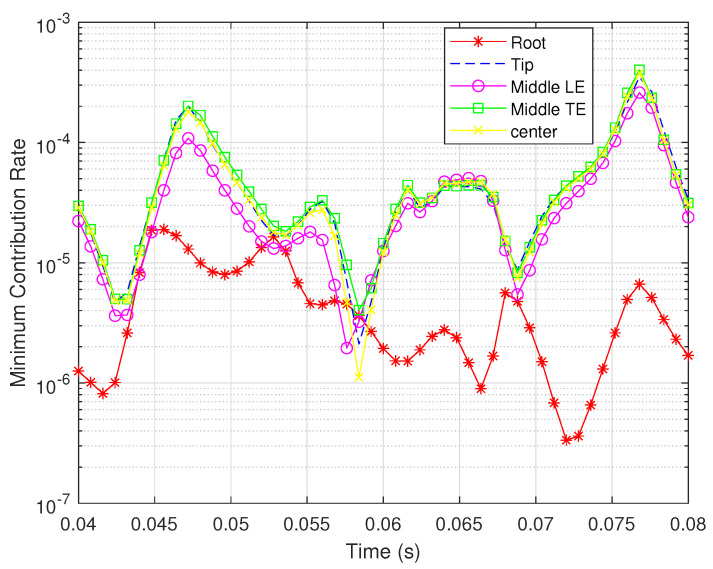
Minimum contribution rates for shear strain sensors located in different places.

**Figure 12:**
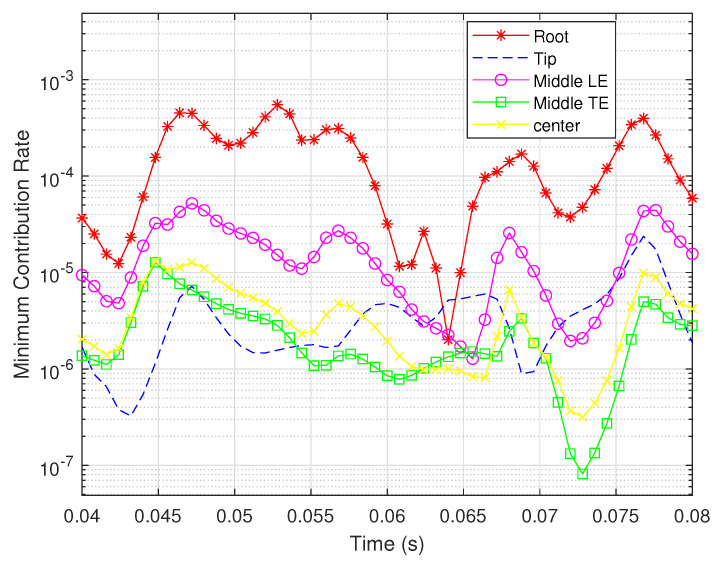
Minimum contribution rates for bending strain sensors located in different places.

Consequently, we chose five typical locations, namely, the root, the tip, the middle of the leading edge, the middle of the trailing edge, and the center of the wing, to show the changes in observability degree during the simulation time. The minimum contribution rates of the rotation rates for the two types of sensors are shown in Figure 11 and Figure 12.

It can be seen that the bending strain sensor at the wing root results in the brawniest observability, while the shear strain sensor at the same place leads to the opposite result. For shear strain sensors, similar observability degrees can be achieved from the other four locations; in particular, the sensors at the tip and the middle of the trailing edge are capable of obtaining the most valuable measurements. However, the effective bending measurements are mainly obtained from the sensor at the root. This result is the same as that demonstrated in Figure 10.

**Figure 13:**
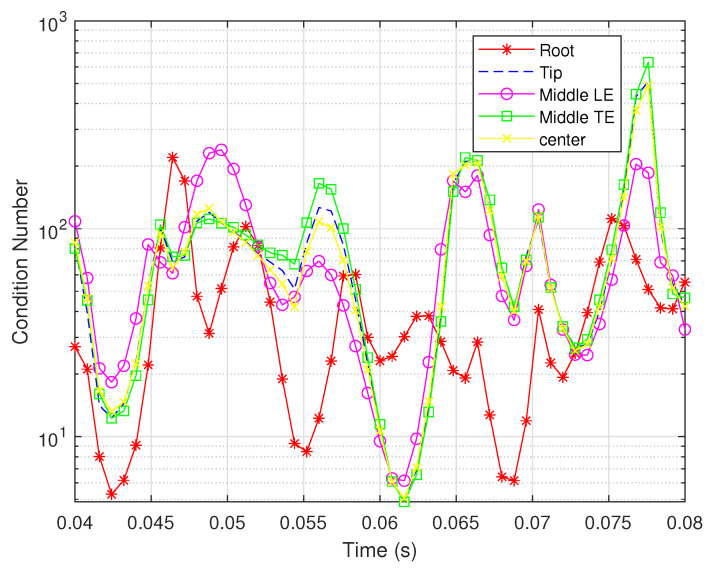
Condition numbers for shear strain sensors located in different places.

**Figure 14:**
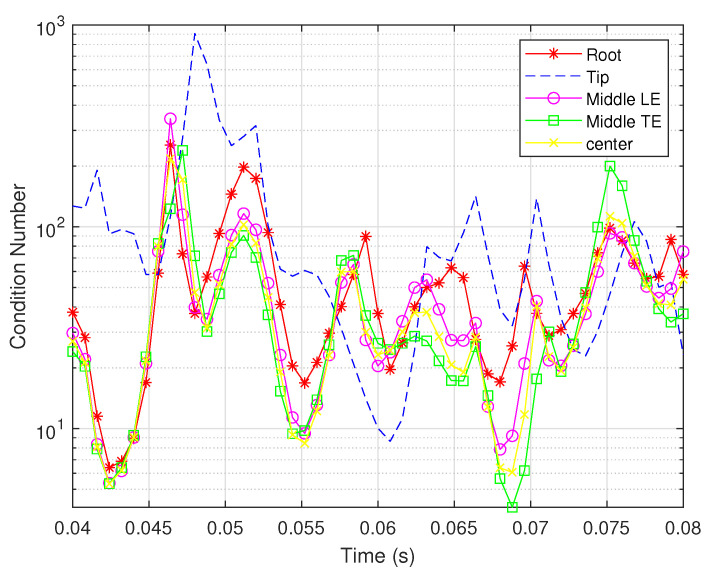
Condition numbers for bending strain sensors located in different places.

The condition numbers of the rotation rates for each sensor location and type are shown in Figure 13 and Figure 14. It can be seen that the condition number for each sensor location and type varies dramatically over time. The observability of the states is primarily more balanced when the shear strain sensors are located at the root and the bending strain sensors are located at the middle of the trailing edge.

**Figure 15:**
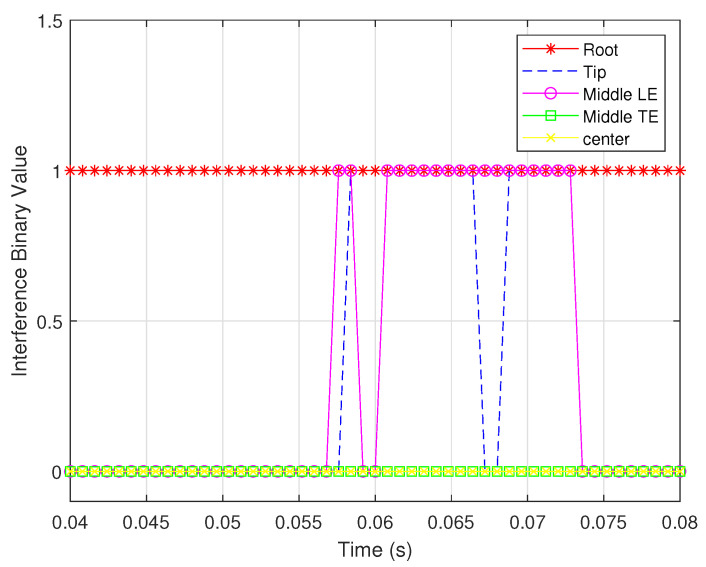
Interference binary values for shear strain sensors located in different places.

**Figure 16:**
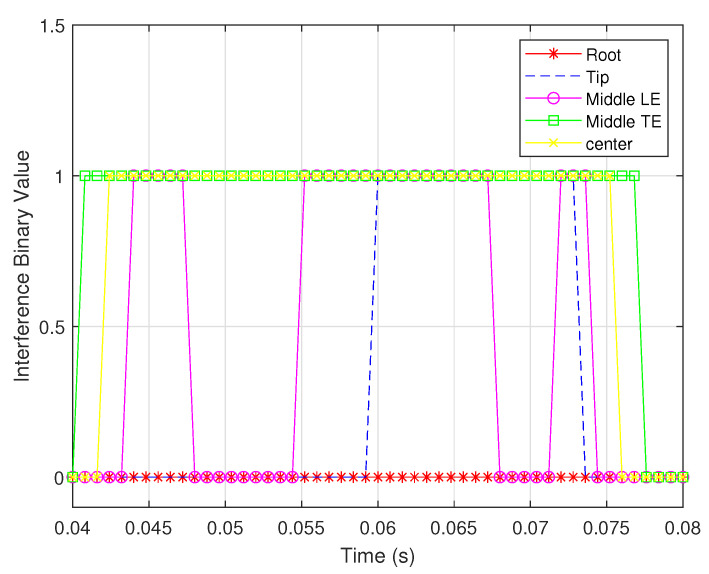
Interference binary values for bending strain sensors located in different places.

When the sensors are assumed to be inaccurate, the noise variances of the shear and bending strain sensors are $1\times 10^{-18}$ and $1\times 10^{-19}$, respectively; the interference binary values from noisy measurements are shown in Figure 15 and Figure 16.

The figures reveal that the observability based on the bending strain measurements is more sensitive to measurement noise than that based on noisy shear strain measurements. Furthermore, for sensors of the same type in different locations, the same measurement noise shows a different effect on the observability. This is because the same sensors at different locations encode different information about the wing rotation rates.

These observability indices provide the main metrics for optimizing the placement of sensors, which is discussed in the next section.

## 6. Observability-Based Optimal Sensor Placement

Here, all sensors are assumed to be located at the wing veins, as this is more consistent with the characteristics of the hawkmoth [24]. The optimal sensor problem is to find the set of sensors that can encode the most information about wing rotation rates based on the proposed observability indices when the possible location and type are given.

As mentioned in Section 4.3, the observability degrees of the rotation rates are more balanced when the condition number is smaller, and the system becomes more observable as the minimum contribution rate becomes larger. In addition, only when the interference binary value equals zero can the negative effect of measurement noise on observability be neglected. Thus, we define the observability-based criterion for the *i*th sensor as $J_{i}$, which is

(64) $$J_{i}=\kappa_{i}+\omega_{\varpi }\varpi \left(\chi_{\min,i}^{1}\right)+\omega_{\Upsilon }\Upsilon_{i}$$

where the normalized condition number of the *i*th sensor is denoted as $\kappa_{i}$, $\chi_{\min,i}^{1}$ stands for the minimum contribution rate of the *i*th sensor, the max-min normalization function is ϖ(x)=(max−x)/(max−min), $\Upsilon_{i}$ is the interference binary value of the *i*th sensor, $\omega_{\varpi }$ and $\omega_{\Upsilon }$ are the weights, and $J_{i}$ shows the *i*th sensor’s ability to encode information about the wing rotation rates, which increases as $J_{i}$ decreases.

To obtain the optimal sensor placement, a set of possible locations of sensors is first determined by sampling the points at every 2 mm along each vein. Then, the observability-based optimal sensor placement problem can be modeled as

(65) $$\begin{array}{cc} \; & \min_{\beta_{i}}\;\;\sum_{i=1}^{p}\beta_{i}J_{i}\\ \; & s.t.\;\left\{\begin{array}{c} \beta_{i}\in 0,1\\ \sum_{i=1}^{p}\beta_{i}=r \end{array}\right. \end{array}$$

where $\beta_{i}$ is a binary activation function, analogous to an off/on switch, and the number of potential sensors in the set p=336, *r* is the desired number of sensors for placement on the wing.

Based on the convex optimization problem and the traversal method, *r* optimal locations out of all potential places on the wing veins are obtained at each time step. The optimal locations change with flexible wing motion and external noise during flight, and thus each possible point’s cumulative number for being an optimal location is counted. A larger cumulative number indicates that a strain sensor placed here is more conducive to improving the observability of the flexible wing flapping system by following the estimation accuracy improvement of rotation rates. The areas where more optimal locations are clustered suggest the most suitable places to arrange the strain sensors.

**Figure 17:**
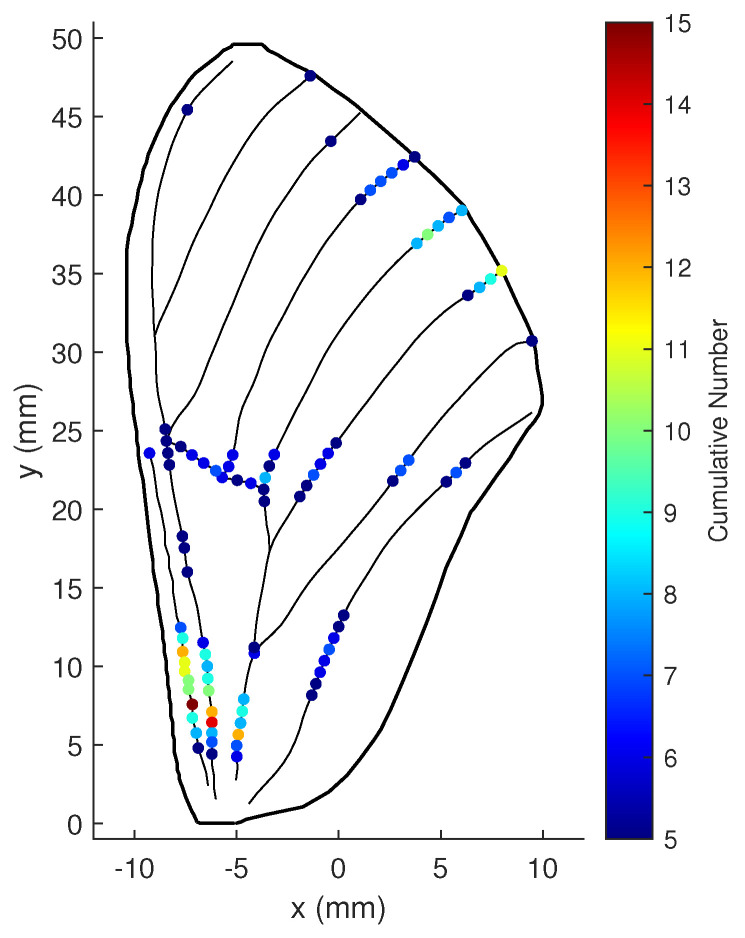
Cumulative numbers for being the optimal location when measurement noise variance is large: $\omega_{\varpi }=5$, $\omega_{\Upsilon }=100$, and r=20.

We assume that the sensors are inaccurate and that measurements inevitably contain noise, as sensors are usually imprecise in practical applications due to technical or external environmental reasons. The noise variances of the shear and bending strain measurements are set as $1\times 10^{-18}$ and $1\times 10^{-19}$, respectively. Let $\omega_{\varpi }$ be 5, $\omega_{\Upsilon }$ be 100, and *r* be 20; then, each possible location’s cumulative number for being an optimal location during the simulation is plotted in Figure 17 (when over 5).

The figure shows that the optimal locations for strain sensors on the wing are divided into three groups: one near the root, one in the center, and one on the upper part of the trailing edge. The wing root is the best location, followed by the upper part of the trailing edge, then the center.

**Figure 18:**
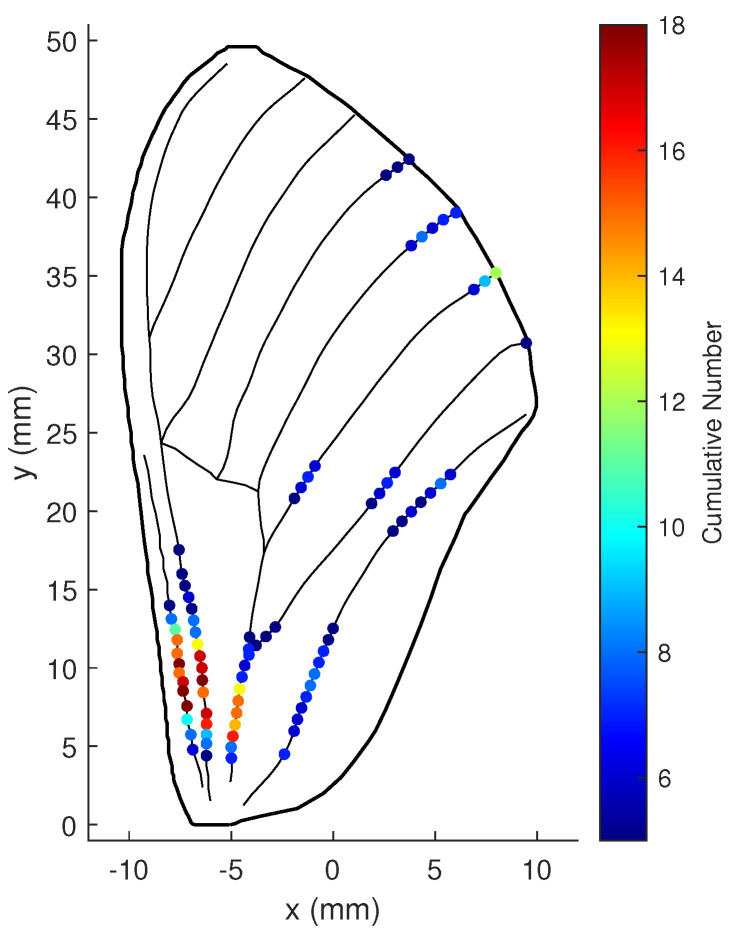
Cumulative numbers for being the optimal location when measurement noise variance is large: $\omega_{\varpi }=1$, $\omega_{\Upsilon }=100$, and r=20.

When the weight of the minimum contribution rate $\omega_{\varpi }$ is reduced to 1, the numerical stability of the states becomes more critical. The cumulative number for each possible location to be optimal in this case is shown in Figure 18. Obviously, more optimal locations are concentrated at the wing root, while the number at the other locations decreases.

**Figure 19:**
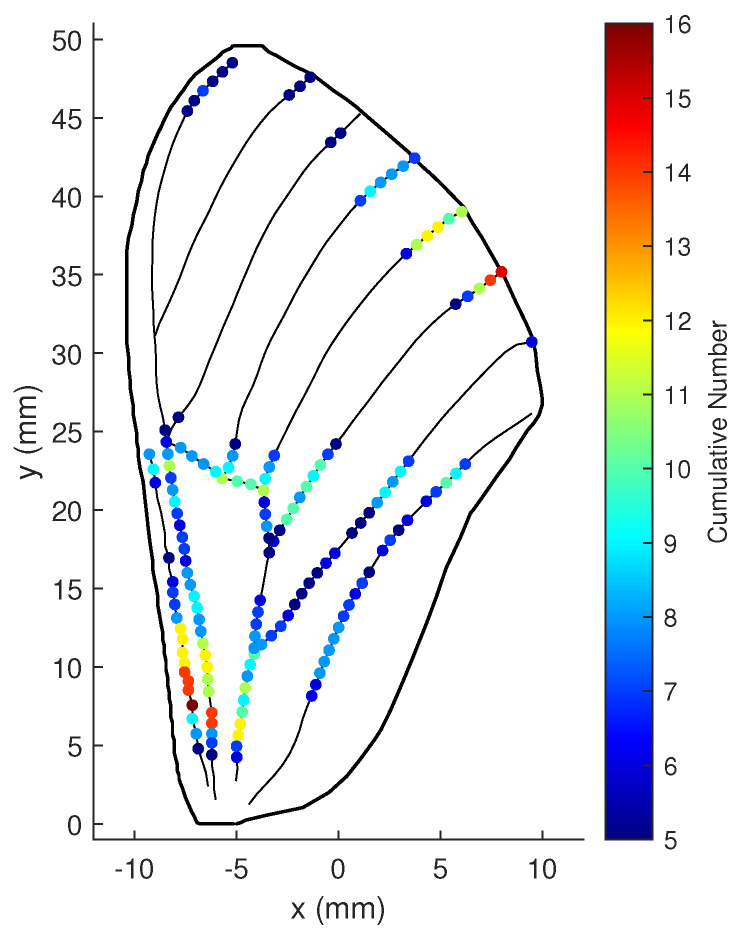
Cumulative numbers for being the optimal location when measurement noise variance is large: $\omega_{\varpi }=5$, $\omega_{\Upsilon }=100$, and r=30.

With r=30 the numbers for all three groups are increased, as illustrated in Figure 19.

**Figure 20:**
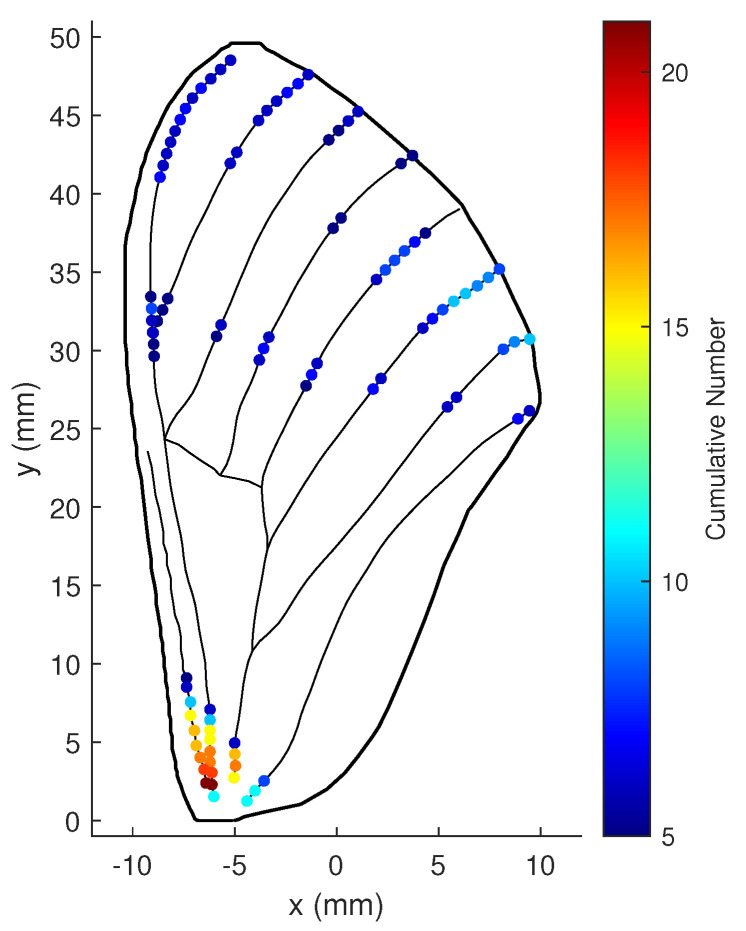
Cumulative numbers for being the optimal location when measurement noise variances is small: $\omega_{\varpi }=5$, $\omega_{\Upsilon }=100$, and r=20.

When the noise variance of the shear and bending strain measurements diminishes to $1\times 10^{-19}$ and $1\times 10^{-20}$, respectively, the best locations on the wing are those shown in Figure 20. In this situation, the numbers for the best locations at the center and upper part of the wing increase.

**Figure 21:**
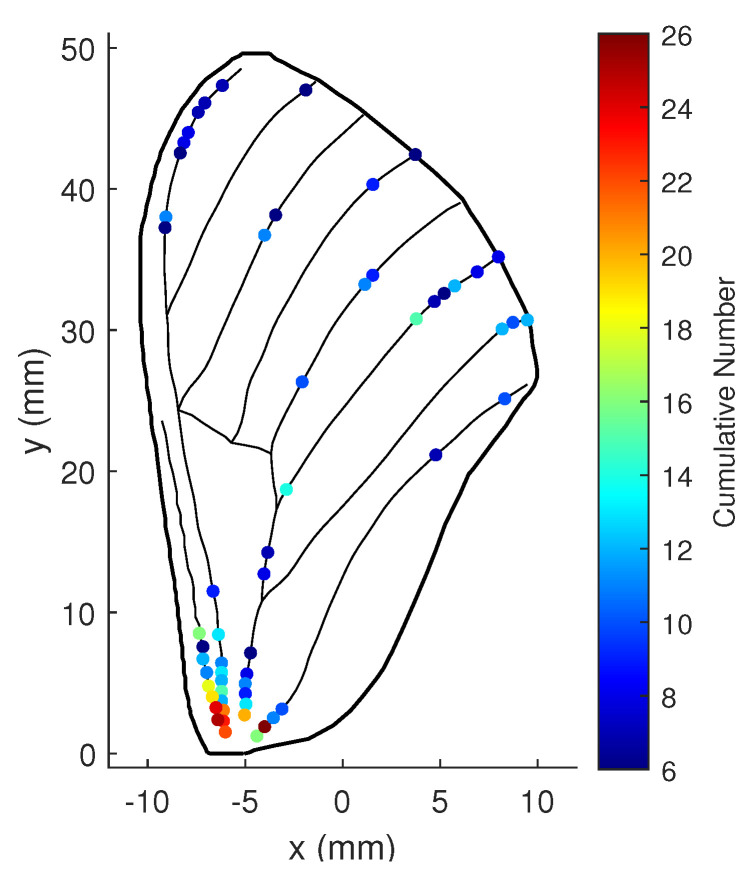
Cumulative numbers for being the optimal location when process and measurement noise variance is large: $\omega_{\varpi }=5$, $\omega_{\Upsilon }=100$, and r=20.

Finally, process noise with the variance of [0.010.010.010.01111] is considered, which is mainly caused by inaccuracy of the dynamic model and unknown control inputs. The best locations on the wing are shown in Figure 21.

The best sensor locations are again clustered into three similar areas, although now most of them are located on the wing root. This is because the observability of the wing system is the brawniest, and the least affected by process noise when the sensors are located at the wing root.

**Figure 22:**
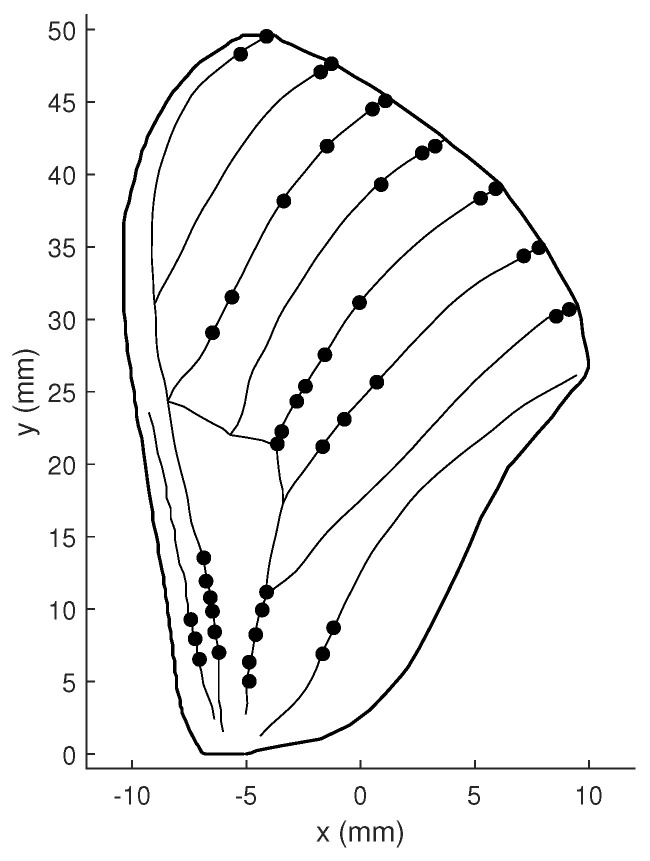
The measured locations of Campaniform Sensilla on the Hawkmoth wing.

Although the optimal sensor placement varies slightly with the different parameter selection, it remains concentrated in three main areas of the wing: one at the wing root, one in the center, and one on the upper part of the trailing edge, which is consistent with the measured locations of the campaniform sensilla on the hawkmoth wing depicted in Figure 22 [24]. The similar optimal placement we obtained based on the gPC-based observability analysis approach proves its validity and practicality.

## 7. Discussion and Conclusions

This paper proposes a derivative-free observability analysis approach based on the generalized Polynomial Chaos expansion for stochastic systems with memory. The observability rank condition provides a binary answer to the observability question, followed by several observability indices to describe the observability degree of the system from different perspectives. For instance, the contribution rate quantifies the observability degree of each state, the condition number presents the numerical stability of all states, and the interference rate measures the influence of measurement noise on the observability. In addition, the equivalence between the traditional and proposed approach for linear systems with memory is proven. The effectiveness of the proposed approach is mathematically demonstrated by applying three different approaches to analysis of the Lorenz system and comparing the results. Sequentially, the proposed approach is utilized to analyze the observability of a flexible wing system and determine the optimal sensor placement. The results show that bending strain sensors should be located at the wing root, while shear strain sensors should be placed at the center and upper part of the wing. The optimal placement we obtained based on the proposed method is similar to the natural distribution of campaniform sensilla on the wing of the hawkmoth.

## Figures and Tables

**Table 1:** The rank of observability matrices calculated via three approaches.

Observability Matrix	Rank
Lie Derivative-Based Observability Matrix	3
Empirical Observability Gramian Matrix	3
gPC-based Observability Coefficient Matrix	6
gPC-based First Order Observability Coefficient Matrix	3

**Table 2:** Computation time of the three approaches.

Approach	Computation Time (s)
Lie Derivative-Based Observability Analysis	0.7940
Empirical Observability Gramian Analysis	87.1700
gPC-based Observability Analysis	1.7980

**Table 3:** Detailed parameters used in the simulation.

Parameter	Symbol	Value	Unit
Wing-Beat Period	$T_{beat}$	40	ms
Memory Length	$t_{M}$	40	ms
Feathering Angle Amplitude	$A_{\alpha }$	45	deg
Position Angle Amplitude	$A_{\zeta }$	60	deg
Mass Density	ρ	220	kg/m${}^{3}$
Chord Length	$c_{wing}$	22	mm
Spanwise Length	$d_{wing}$	50	mm
Leading Edge Thickness	$t_{RO}$	0.26	mm
Root Thickness	$t_{RO}$	0.26	mm
Decay Rate from LE to TE	$a_{LE}$	0.24	−
Decay Rate from Root to Tip	$a_{RO}$	0.24	−
Mode Frequency of First Bending Mode	$f_{1}$	50	Hz
Mode Frequency of First Torsion Mode	$f_{2}$	55	Hz
Rotation Axis	$x_{r}$	−4.68	mm
Air Fluid Density	$\rho_{f}$	1.225	kg/m${}^{3}$
Delay of STA Function	*a*	5	ms
Width of STA Function	*b*	4	ms
STA Frequency	$f_{STA}$	159.155	Hz
Slope of NLA Function	*c*	0.15	−
Half-Maximum position of NLA Function	*d*	0.005	−
Normalization Constant	$C_{\varkappa }$	$1.084\times 10^{-4}$	−

## Data Availability

Not applicable.

## References

[1] Mishra S., Tripathi B., Garg S., Kumar A., Kumar P. (2015). Design and development of a bio-inspired flapping wing type micro air vehicle. Procedia Mater. Sci..

[2] Van Truong T., Nguyen Q.V., Lee H.P. (2017). Bio-inspired flexible flapping wings with elastic deformation. Aerospace.

[3] Bhatti M.Y., Lee S.G., Han J.H. (2021). Dynamic Stability and Flight Control of Biomimetic Flapping-Wing Micro Air Vehicle. Aerospace.

[4] Gollisch T., Meister M. (2010). Eye smarter than scientists believed: Neural computations in circuits of the retina. Neuron.

[5] Fox J.L., Fairhall A.L., Daniel T.L. (2010). Encoding properties of haltere neurons enable motion feature detection in a biological gyroscope. Proc. Natl. Acad. Sci. USA.

[6] Pratt B., Deora T., Mohren T., Daniel T. (2017). Neural evidence supports a dual sensory-motor role for insect wings. Proc. R. Soc. Biol. Sci. USA.

[7] Boyacioglu B., Morgansen K.A. Bioinspired observability analysis tools for deterministic systems with memory in flight applications. Proceedings of the AIAA Scitech 2021 Forum.

[8] Hinson B.T., Morgansen K.A. (2015). Gyroscopic sensing in the wings of the hawkmoth Manduca sexta: The role of sensor location and directional sensitivity. Bioinspir. Biomim..

[9] Chen C.T. (1984). Linear System Theory and Design.

[10] Rouhani A., Abur A. (2016). Observability analysis for dynamic state estimation of synchronous machines. IEEE Trans. Power Syst..

[11] Seo M.G., Tahk M.J. (2015). Observability analysis and enhancement of radome aberration estimation with line-of-sight angle-only measurement. IEEE Trans. Aerosp. Electron. Syst..

[12] Krener A.J., Ide K. Measures of unobservability. Proceedings of the 48h IEEE Conference on Decision and Control (CDC) Held Jointly with 2009 28th Chinese Control Conference.

[13] Hodzic E., Morgansen K.A. Simulation-Based Observability Analysis Tools for Experimental Aerospace Applications. Proceedings of the 2021 American Control Conference (ACC).

[14] Hinson B.T., Binder M.K., Morgansen K.A. Path planning to optimize observability in a planar uniform flow field. Proceedings of the 2013 American Control Conference.

[15] Zheng Z., Xu Y., Mili L., Liu Z., Peng L., Wang Y. (2021). Derivative-free observability analysis of a stochastic dynamical system. IEEE Trans. Netw. Sci. Eng..

[16] Zheng Z., Xu Y., Mili L., Liu Z., Korkali M., Wang Y. (2021). Observability analysis of a power system stochastic dynamic model using a derivative-free approach. IEEE Trans. Power Syst..

[17] Mohren T.L., Daniel T.L., Brunton S.L., Brunton B.W. (2018). Neural-inspired sensors enable sparse, efficient classification of spatiotemporal data. Proc. Natl. Acad. Sci. USA.

[18] Krantz S.G., Parks H.R. (2002). The Implicit Function Theorem: History, Theory, and Applications.

[19] Ren Z., Li W., Billinton R., Yan W. (2015). Probabilistic power flow analysis based on the stochastic response surface method. IEEE Trans. Power Syst..

[20] Xiu D., Karniadakis G.E. (2002). The Wiener–Askey polynomial chaos for stochastic differential equations. Siam J. Sci. Comput..

[21] Willmott A.P., Ellington C.P. (1997). The mechanics of flight in the hawkmoth Manduca sexta. I. Kinematics of hovering and forward flight. J. Exp. Biol..

[22] Hedrick T.L., Daniel T.L. (2006). Flight control in the hawkmoth Manduca sexta: The inverse problem of hovering. J. Exp. Biol..

[23] Reade J., Jankauski M. (2022). Investigation of chordwise functionally graded flexural rigidity in flapping wings using a two-dimensional pitch-plunge model. Bioinspir. Biomim..

[24] Dickerson B.H., Aldworth Z.N., Daniel T.L. (2014). Control of moth flight posture is mediated by wing mechanosensory feedback. J. Exp. Biol..

